# Analgesic Self-Medication and Safety Knowledge Among Adults in the United Arab Emirates: A Cross-Sectional Online Survey

**DOI:** 10.3390/healthcare14213655

**Published:** 2026-10-28

**Authors:** Eslam ElNebrisi, Ayesha Amal, Raya Zahid Ahmad, Sarah Fadhil Hussein Ghayb Al-Rubaye, Anam Mansoor, Maya Ahmed, Haya Al Shawa

**Affiliations:** 1 Basic Sciences Department, College of Medicine, RAK Medical and Health Sciences University, Ras Al Khaimah P.O. Box 11172, United Arab Emirates; 2 Department of Biomedical Sciences, Dubai Medical College for Girls, Dubai Medical University, Dubai P.O. Box 20170, United Arab Emirates; anm20190108@dmu.ae; 3 Department of Internal Medicine, Dubai Medical College for Girls, Dubai Medical University, Dubai P.O. Box 20170, United Arab Emirates; aym20190113@dmu.ae (A.A.); rza20190149@dmu.ae (R.Z.A.); sfh20190159@dmu.ae (S.F.H.G.A.-R.); mam20190139@dmu.ae (M.A.); 4 College of Nursing, Dubai Medical University, Dubai P.O. Box 20170, United Arab Emirates

**Keywords:** self-medication, analgesics, paracetamol, non-steroidal anti-inflammatory drugs, medication safety, health education, community pharmacy, cross-sectional studies

## Abstract

**Highlights:**

**What are the main findings?**

• Most participants (83.0%) reported having ever self-medicated with analgesics, most commonly paracetamol (93.7% of self-medicators), and sociodemographic factors explained little of the variation in this practice.
• Among self-medicators, safety knowledge was uneven: hepatic and gastrointestinal risks were widely recognized, whereas paracetamol allergic reactions (47.3%), NSAID-related renal risk (56.0%) and paracetamol toxicity at high doses (67.5%) were less often recognized, and 14.8% reported double dosing when symptoms persisted.

**What are the implications of the main findings?**

• The specific gaps identified (allergic and renal risks, not exceeding the recommended dose, and what to do when a first dose fails) suggest content for future educational messages, whose usefulness requires evaluation.
• Because self-medication was reported across all sociodemographic groups in this sample, point-of-sale approaches may be worth exploring; this requires confirmation in representative samples and evaluation in intervention studies.

**Abstract:**

**Background/Objectives:** Analgesic self-medication is common worldwide, yet unsupervised use carries hepatic, gastrointestinal, and renal risks when safety knowledge is incomplete, and community data from the United Arab Emirates (UAE) are limited. This study described lifetime self-reported analgesic self-medication, its sociodemographic correlates, and knowledge of paracetamol and non-steroidal anti-inflammatory drug (NSAID) safety in an online convenience sample of adults in the UAE. **Methods:** A cross-sectional online survey (April–October 2024) used convenience sampling and a self-administered English/Arabic questionnaire. Only self-medicators completed a study-specific five-item safety knowledge index. Analyses included chi-square, Mann–Whitney U and exploratory regression. **Results:** Of 400 participants (65.5% female), 332 (83.0%) reported lifetime self-medication with analgesics, mostly paracetamol (93.7%) and ibuprofen (58.7%). Among self-medicators, mean knowledge was 3.12/5: hepatotoxicity (71.1%) and gastrointestinal risk (70.2%) were widely recognized, whereas renal risk (56.0%) and paracetamol allergy (47.3%) were less so; 14.8% reported double dosing when symptoms persisted. After adjustment, female sex was associated with self-medication (OR 2.08, 95% CI 1.12–3.86) in a marginally significant model, and female sex (B = 0.55, 95% CI 0.15–0.95) and younger age (B = −0.027 per year, 95% CI −0.041 to −0.012) were associated with higher knowledge scores. **Conclusions:** In this online convenience sample, safety knowledge (as measured by a brief study-specific index) was selective rather than uniformly limited. Education addressing allergic and renal risks, not exceeding recommended doses, and what to do when a first dose fails may be useful but requires evaluation in intervention studies.

## 1. Introduction

Self-medication, broadly defined as the use of medicinal products without professional medical supervision, is a public health concern with implications for patient safety and healthcare systems. Prevalence estimates range from 11.7% to over 90%, depending on the population and setting [1,2]. Self-medication is more common in some population groups. For example, studies report that 12–68% of pregnant women engage in self-medication, often due to limited awareness of medication risks during pregnancy [3,4]. Among university students, prevalence as high as 73% has been reported, primarily attributed to the accessibility of over-the-counter (OTC) medications and a tendency to manage minor ailments independently [2,5,6,7,8]. During the COVID-19 pandemic, an estimated 25–65% of the global population engaged in self-medication to prevent or manage symptoms without consulting healthcare professionals [9,10]. Among elderly individuals, self-medication prevalence ranges from 33% to 50%, often influenced by long-standing familiarity with medications and a desire to manage recurring health issues autonomously [11,12,13].

Analgesics are among the most commonly used medications in self-medication, especially for mild conditions such as headaches, musculoskeletal pain, and fever [14]. Paracetamol (acetaminophen) is widely regarded as a first-line agent due to its efficacy and safety profile when used correctly [15,16]. Studies show its frequent use across diverse populations, including among patients with chronic conditions such as ulcerative colitis [17]. Similarly, NSAIDs such as ibuprofen, diclofenac, and aspirin are widely available and frequently used without prescription [18,19]. However, unsupervised use and prolonged use of analgesics carries health risks; NSAIDs, for instance, are associated with gastrointestinal bleeding, renal impairment, and cardiovascular events [18,19,20,21]. Paracetamol overdose remains a leading cause of drug-induced liver injury globally [22]. Despite these risks, public knowledge regarding the safe use of these medications remains inconsistent. Prior studies have reported widespread gaps in awareness, even among relatively well-educated populations.

Self-medication has increasingly been studied in the Middle East. While self-medication and over-the-counter analgesic use have been reported in the UAE, fewer studies have specifically examined analgesic-related safety knowledge and potentially unsafe self-management responses among community adults. Most regional studies have focused on specific subgroups, such as university students [7] or healthcare professionals, limiting the generalizability of findings to the broader population. One UAE-based study reported that approximately 60% of university students used OTC medications without a prescription, including analgesics [7]. However, there remains limited evidence focusing specifically on analgesic safety knowledge and behavioral responses to self-medication at the community level. This gap is relevant because these medicines are easily available and can be misused.

Designing effective medication-safety education requires knowing which specific safety concepts the public already holds and which it does not. Broad awareness campaigns are unlikely to change behavior if they reinforce risks that are already widely recognized while omitting those that are not. The present study therefore examined self-reported analgesic self-medication among adults in the UAE, the sociodemographic factors associated with it, and, as the primary educational target, the distribution of specific safety knowledge among participants who reported self-medication. The findings are intended to generate hypotheses about the content and delivery of community-level medication-safety education.

## 2. Materials and Methods

### 2.1. Study Design and Setting

A descriptive cross-sectional study was conducted in the United Arab Emirates (UAE) between April and October 2024 to assess self-medication practices involving analgesics among community-dwelling adults. Data were collected using an online self-administered questionnaire (Google Forms; Google LLC, Mountain View, CA, USA) available in English and Arabic to improve accessibility. The questionnaire was designed to be completed in approximately 3–5 min, as stated on the participant information and consent page presented before survey initiation.

The study aimed to describe the proportion of participants reporting lifetime analgesic self-medication, factors influencing this practice, and their knowledge regarding analgesic safety.

The questionnaire was reviewed by experts and piloted before fielding (Section 2.3). The technical functionality of the electronic form, including item display, branching logic, and response capture, was verified before dissemination. Reporting follows the STROBE statement for cross-sectional studies and the CHERRIES checklist for web-based surveys (Supplementary Materials S1 and S2).

### 2.2. Study Participants and Sampling

The study targeted adults aged 18 years and older residing in the UAE. Individuals of any sex or educational background were eligible to participate. The study excluded participants currently receiving prescribed analgesic therapy under direct medical supervision, individuals younger than 18 years, and temporary visitors or tourists.

A convenience sampling strategy was employed. The questionnaire link was shared through social media platforms, including WhatsApp, Facebook, Twitter/X, and Snapchat, as well as institutional and professional communication channels. Participation was entirely voluntary and anonymous. Before accessing the survey questions, participants were presented with an electronic informed consent form explaining the purpose of the study, confidentiality measures, and their right to withdraw at any stage prior to submission.

The study was conducted exclusively in the United Arab Emirates, and participants were recruited from within the UAE through online platforms. Given the use of a non-probability convenience sampling approach, the study was not designed to produce population-representative estimates, but rather to provide exploratory insights into self-medication practices and knowledge patterns within a community-based sample.

Because the survey was self-administered online, eligibility was assessed through self-report. The participant information page, presented before consent, stated the eligibility criteria and asked individuals not to proceed if they were younger than 18 years, were visitors or tourists, or were currently receiving prescribed analgesic therapy under direct medical supervision; participants confirmed that they had read this information before accessing the questionnaire, and age was additionally recorded within the questionnaire. Residency status and current prescribed therapy could not be independently verified.

### 2.3. Survey Instrument

A structured questionnaire was developed for this study based on a review of the relevant literature and pilot-tested prior to dissemination. The English and Arabic versions are provided as Supplementary Material S3. The instrument comprised three sections:

• Sociodemographic characteristics: age (collected in categories), sex (recorded as binary self-reported sex: male/female), and education level.
• Self-medication practices: whether the participant self-prescribes analgesics, the types used (paracetamol and specific NSAIDs), the symptoms or conditions prompting use, the motivations influencing the decision to self-medicate, the factors influencing analgesic selection, and the actions taken when self-medication failed to relieve symptoms. Items on concomitant medication and polytherapy were not included.
• Analgesic safety knowledge: five statements addressing high-priority safety concepts for paracetamol and NSAIDs (gastrointestinal effects, renal risk, hepatotoxicity, toxicity at high doses, and allergic reactions), to which participants responded “True,” “False,” or “Not sure.” Items were selected to capture priority safety risks rather than to provide a comprehensive assessment of analgesic safety literacy.

On completion of the questionnaire, all participants were presented with a downloadable digital brochure summarizing analgesic safety information (Supplementary Material S4). The brochure addressed paracetamol hepatotoxicity, NSAID-related gastrointestinal bleeding, maximum daily doses and safe-use practices; it did not cover renal risk or allergic reactions. No post-exposure assessment was conducted, and the brochure is described here as a component of the study design rather than as an evaluated intervention.

The questionnaire was developed in English and translated into Arabic by a bilingual member of the research team. The Arabic version was then back-translated into English by a second bilingual individual who was not involved in the forward translation, and the two English versions were compared by the research team. Participants chose the language in which to complete the survey; 326 (81.5%) completed the English version and 74 (18.5%) the Arabic version. The English and Arabic versions differed in two response options for age: the Arabic version listed ‘45–55’ where the English version listed ‘45–54’, and ‘more than 65’ where the English version listed ‘65 and above’. Both discrepancies affected only boundary ages. For analysis, responses were harmonized to the English categories, and the upper categories were merged into a single ≥55-year group. Only three respondents selected either option, so we judged the impact on comparability to be negligible. In the Arabic version, knowledge item 2 was phrased negatively (“NSAIDs are generally not safe for individuals with kidney problems”); Arabic-version responses to this item were therefore scored with “True” as the correct answer, so that all scores reflect the same safety concept.

Analgesic safety knowledge was assessed using a study-specific five-item index developed by the research team for this survey. Items were based on established safety information for paracetamol and NSAIDs, covering the gastrointestinal [23] and renal [24] risks of NSAIDs, and the dose-related hepatotoxicity [25] and hypersensitivity reactions [26] of paracetamol. Before data collection, the questionnaire was reviewed for content relevance and clarity by three experts in pharmacology, pharmacotherapy and community pharmacy practice, and their suggestions were incorporated. It was then piloted with eight participants to check the clarity, wording and completion time of the items; this pilot assessed feasibility only and was not intended as a validation of the instrument. Pilot responses were excluded from the analysis. The index was intended as a brief, exploratory measure of specific safety concepts rather than a comprehensive or formally validated assessment of analgesic knowledge.

Each knowledge item was scored dichotomously (1 = correct; 0 = incorrect or “Not sure”), and the five items were summed to produce a composite index ranging from 0 to 5. “Not sure” responses were scored as incorrect on the basis that uncertainty about a safety concept does not confer protective behavior, although this decision does not distinguish between misconception and absence of knowledge. We evaluated the index’s psychometric properties post hoc (internal consistency, corrected item–total correlations, item discrimination, inter-item correlations, split-half reliability, and principal component analysis) and found acceptable internal consistency (Cronbach’s α = 0.713) and a predominantly unidimensional structure on principal component analysis. Full results are reported in Supplementary Material S5. Principal component analysis was used descriptively to check whether the five items could reasonably be summed into a single score, and not to establish construct validity. Because Pearson correlations between binary items can understate their association, the analysis was repeated using tetrachoric correlations, which gave the same single-component result.

The first primary outcome was lifetime self-reported analgesic self-medication, assessed with a single item: “Have you ever engaged in self-prescription (taking medication without consulting a healthcare professional) of pain medication?” with response options “Yes,” “No,” and “Not sure.” An operational definition was presented to participants within the item stem, and the term “self-prescription” was used in the questionnaire to denote the same construct reported here as self-medication. Because the item used an “ever” framing, with no defined recall period, this measure reflects lifetime self-reported practice and is not directly comparable with studies reporting 12-month or point prevalence. Participants responding “Yes” then completed the subsequent items on the analgesics used, indications, motivations, selection criteria, and actions taken when symptom relief was not achieved, as well as the five analgesic safety knowledge items.

### 2.4. Sample Size

The minimum required sample size was estimated using the standard formula for a single proportion, *n* = Z^2^*p*(1 − *p*)/d^2^, assuming a conservative expected proportion of 50% (*p* = 0.5, maximizing required sample size), a 95% confidence level (Z = 1.96), and a 5% margin of error (d = 0.05). This yielded a minimum of 384 participants. Given the size of the adult population of the United Arab Emirates, no finite population correction was applied. A total of 400 complete responses were obtained, exceeding this requirement. This calculation was based on the descriptive estimate of lifetime self-medication only; the study was not powered a priori for the regression models, which were therefore treated as exploratory. As a post hoc check of their adequacy, the logistic model for self-medication (Model 1) had approximately 23 outcome events per predictor (68 participants who did not self-medicate; three predictors), and the logistic model for adequate knowledge (Model 3) had approximately 54 (162 participants with adequate knowledge), both above the commonly recommended minimum of 10 events per predictor. For the linear model (Model 2), the sample of 332 self-medicating participants provided 80% power at a two-sided α of 0.05 to detect a small effect of a single predictor (f^2^ = 0.024, equivalent to about 2.3% of variance explained) in a model with three predictors.

### 2.5. Study Outcomes

The primary outcomes were: (i) lifetime self-reported analgesic self-medication (Section 2.3), and (ii) the level of analgesic safety knowledge among self-medicating participants assessed using a brief five-item knowledge index.

Secondary outcomes were: (i) the types of analgesics used; (ii) the symptoms or conditions prompting self-medication; (iii) motivations for self-medication and factors influencing analgesic selection; (iv) actions taken when self-medication was unsuccessful; (v) associations between sociodemographic characteristics and both self-medication practices and knowledge index scores.

### 2.6. Data Analysis

Data were screened for completeness and potential duplicate entries before analysis; no duplicates were identified. Analyses were performed using IBM SPSS Statistics version 28.0 (IBM Corp., Armonk, NY, USA) and Python version 3.11 (Python Software Foundation, Wilmington, DE, USA) with the statsmodels (version 0.15) and SciPy (version 1.17) libraries; Figures 2 and 3 were produced with Matplotlib (version 3.10). Categorical variables (e.g., sex, engagement in self-medication, types of analgesics used, reasons for self-medication, and knowledge responses) were summarised as frequencies and percentages, and count variables (number of items endorsed) as mean and standard deviation (SD).

Bivariate associations between self-medication and participant characteristics were examined using chi-square tests for independence with continuity correction for 2 × 2 tables; Fisher’s exact test was used where expected cell counts were below five. Cramér’s V was calculated as a measure of effect size for categorical associations, and Mann–Whitney U tests compared knowledge index scores between groups. An exploratory analysis used a dichotomised age variable (18–34 vs. ≥35 years) to examine age-related differences in individual knowledge items. Because knowledge index scores were not normally distributed, they were summarised as median and interquartile range (IQR) when compared between groups.

Because the sample size was calculated for the descriptive outcome only (Section 2.4), the multivariable analyses were exploratory and are interpreted as such. Three multivariable models were fitted with sex (0 = male, 1 = female), age and educational level (ordinal: 1 = primary, 2 = secondary, 3 = university or above) entered simultaneously as predictors: binary logistic regression for self-medication (Model 1, *n* = 400), multiple linear regression for the knowledge index score (Model 2, *n* = 332), and binary logistic regression for adequate knowledge, defined as at least four of five items correct (Model 3, *n* = 332). This cut-off corresponds to 80% correct responses, the threshold for good knowledge under Bloom’s cut-off criteria, which are widely used in knowledge, attitude and practice surveys [27]. Because no externally validated threshold is available for this index, the ≥4/5 categorization is an exploratory operational definition rather than a clinically validated standard of adequate knowledge. The continuous score (Model 2) was therefore treated as the primary knowledge outcome, and Model 3 was re-fitted using alternative cut-offs of ≥3/5 and 5/5. Because only two participants reported primary education, a three-category coding of education could not be estimated reliably; the models were therefore also re-fitted with primary and secondary education combined, giving a binary variable (university vs. school education), to confirm that the ordinal coding, which assumes equal intervals between levels, did not affect the estimates. Sex, age and education were selected a priori as covariates because they were the sociodemographic characteristics collected in the survey and are among the factors most consistently associated with self-medication in previous studies [1,2]. Unadjusted estimates from single-predictor models are reported alongside adjusted estimates, and, to examine the change in the estimate for sex in Model 1, age and education were also added separately (Supplementary Material S6). Model fit was assessed using the Nagelkerke R^2^ and Hosmer–Lemeshow test for logistic models and the adjusted R^2^ for the linear model, and a sex × age interaction term was tested in Model 2.

Age was entered as a continuous variable using category midpoints, an approximation that assumes ages are evenly distributed within each category. This approach was chosen to preserve statistical power: only 68 respondents reported no self-medication, and entering age as four indicator variables would have reduced the number of outcome events per model parameter from about 23 to about 11. To test whether this approximation affected the estimates, all models were re-fitted with age entered as an ordinal score (1–5), as categorical indicator variables with 18–24 years as the reference, and as a binary variable (18–34 vs. ≥35 years), and model fit was compared using likelihood-ratio and F tests. Full estimates from the categorical models are reported in Supplementary Material S6. To assess whether the language version affected the findings, all models were also re-fitted with questionnaire language (Arabic vs. English) as an additional covariate, and internal consistency of the knowledge index was calculated separately for each version (Supplementary Material S6).

All significance tests were two-tailed, with statistical significance set at *p* < 0.05. The study was designed primarily to describe lifetime self-medication and the distribution of safety knowledge, and these descriptive estimates are the primary results. All hypothesis tests, including the bivariate comparisons, the item-level comparisons by sex and age group, and the regression models, were exploratory, and the *p* values reported are not adjusted for multiple comparisons. As a sensitivity analysis, the Holm–Bonferroni correction was applied across the ten item-level comparisons by sex and age group (five items × two groupings).

### 2.7. Ethical Considerations

This study received ethical approval from the Research and Ethics Committee of Dubai Medical University (Approval Reference No. REC/DMCG/AY23-24/F 3). Participation was entirely voluntary. The online consent form at the start of the survey informed participants about the study’s purpose, assured them of the confidentiality of their responses, and emphasized that they could exit the questionnaire at any point before submission. No personal identifying information was collected in the survey, and all data were analyzed in aggregate. Response data were securely stored on a password-protected device accessible only to the principal investigator. Participants were not exposed to any interventions or risks beyond those of everyday online activities. All methods were carried out in accordance with relevant guidelines and regulations for research with human subjects.

## 3. Results

### 3.1. Sociodemographic Characteristics of Participants

**Figure 1:**
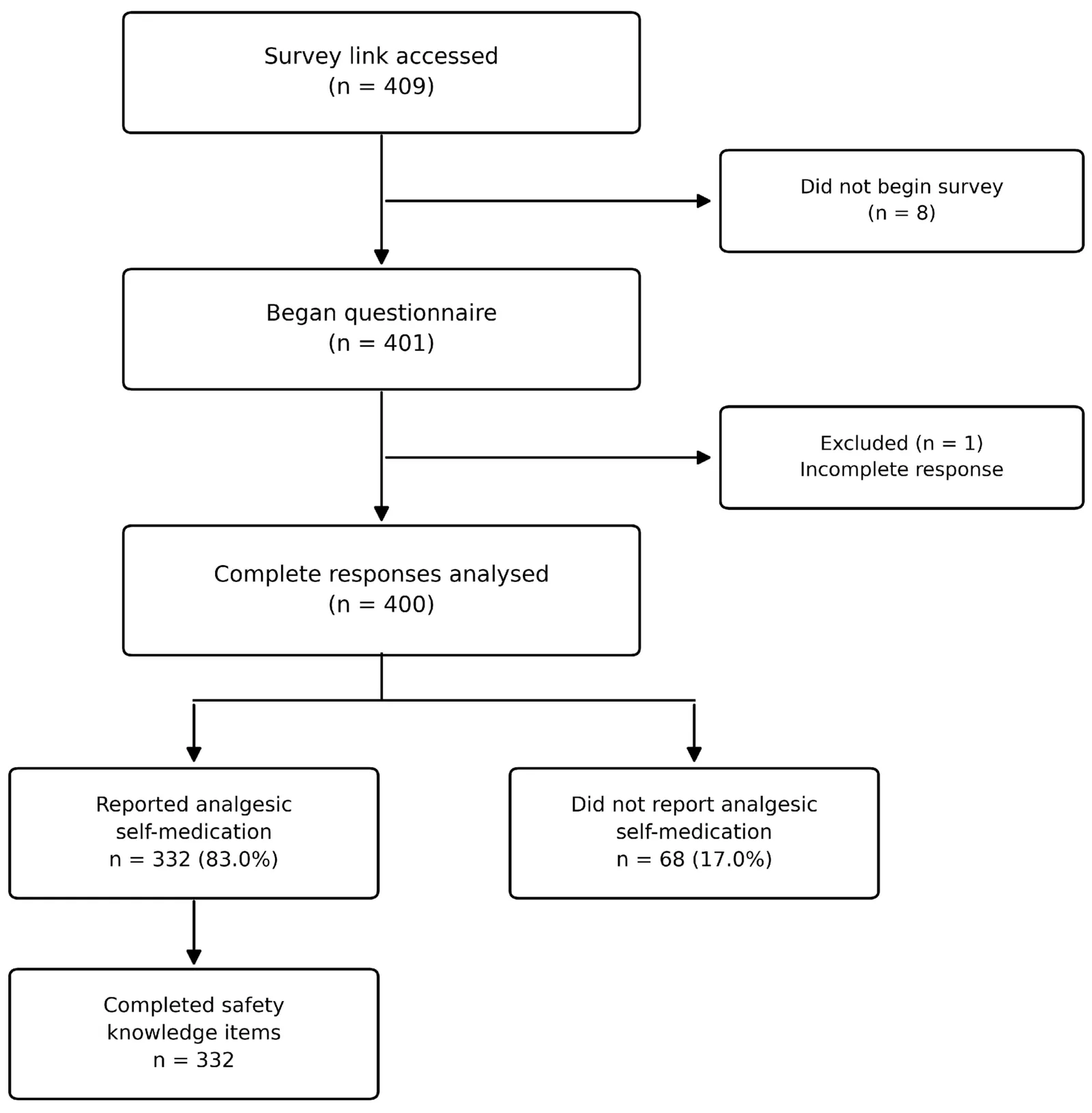
Flow of participants through the study. The survey link was accessed 409 times; 401 questionnaires were started and 400 were submitted complete. Knowledge items were completed only by participants reporting analgesic self-medication (*n* = 332); safety knowledge results therefore describe this subsample.

The survey link was accessed 409 times; 401 questionnaires were started, 400 were submitted complete, and one incomplete response was excluded (Figure 1). Because the survey was distributed as an open link, accesses may not represent unique or eligible individuals, so these figures describe participant flow rather than a survey response or participation rate. The 400 complete questionnaires were screened for eligibility using the recorded age item and participants’ confirmation of the information page, which stated the eligibility criteria. No participants were excluded on eligibility grounds. Females accounted for 65.5% (*n* = 262) of the sample and males for 34.5% (*n* = 138). The most represented age category was 18–24 years (36.0%, *n* = 144), followed by 35–44 years (25.8%, *n* = 103), 45–54 years (15.0%, *n* = 60), 25–34 years (14.5%, *n* = 58), and ≥55 years (8.8%, *n* = 35). Most participants had university-level education or higher (84.8%, *n* = 339), while 14.8% (*n* = 59) had completed secondary education and 0.5% (*n* = 2) primary education. Detailed characteristics are presented in Table 1.

### 3.2. Self-Reported Self-Medication Practices and Types of Analgesics Used

Among the 400 respondents, 83.0% (*n* = 332) reported lifetime self-medication with analgesics (having ever self-medicated), while 17.0% (*n* = 68) reported never having done so.

Paracetamol was the most frequently used analgesic among self-medicating participants, reported by 93.7% (*n* = 311), followed by ibuprofen (58.7%, *n* = 195) and diclofenac (39.2%, *n* = 130). Other analgesics included mefenamic acid (17.8%, *n* = 59) and aspirin (15.4%, *n* = 51).

Headache was the leading symptom prompting self-medication (77.1%, *n* = 256), followed by fever (53.3%, *n* = 177), menstrual pain (35.5%, *n* = 118), muscular pain (31.6%, *n* = 105), and dental pain (28.6%, *n* = 95). Other reported symptoms included runny nose, joint pain, cough, diarrhea, and post-surgical pain.

Among self-medicating participants, the mean number of analgesics used was 2.24 (SD = 1.11). Participants reported a mean of 3.12 (SD = 2.04) symptoms associated with self-medication practices. Detailed findings are summarized in Table 2.

### 3.3. Motivations for Self-Medication and Factors Influencing Analgesic Choice

Participants identified several motivations for practicing self-medication. The most frequently reported reasons were previous positive experiences with analgesics (58.1%, *n* = 193), convenience (57.8%, *n* = 192), and perceiving the illness as mild (57.2%, *n* = 190). Other contributing factors included cost-effectiveness (34.3%, *n* = 114), lack of time to visit healthcare professionals (33.1%, *n* = 110), and lack of health insurance or financial difficulties (11.4%, *n* = 38). Participants reported a mean of 2.52 (SD = 1.37) motivations for self-medication practices.

When selecting analgesics, previous effectiveness in pain relief was the strongest influencing factor (78.0%, *n* = 259), followed by previous physician recommendation (48.8%, *n* = 162) and ease of accessibility (47.6%, *n* = 158). Affordability and concerns regarding side effects of alternative medications were also reported. Participants identified a mean of 2.22 (SD = 1.15) factors influencing analgesic selection.

When self-medication failed to relieve symptoms, 60.2% (*n* = 200) of participants reported seeking medical consultation. Other actions included waiting for symptoms to resolve spontaneously (31.0%, *n* = 103), switching to another analgesic (29.8%, *n* = 99), using home remedies or alternative medicine (21.7%, *n* = 72), double dosing (14.8%, *n* = 49), and seeking advice from friends or family members (13.3%, *n* = 44). Participants reported a mean of 1.71 (SD = 0.96) actions following unsuccessful self-medication attempts and use. Detailed findings are presented in Table 3.

### 3.4. Knowledge Regarding Analgesic Safety Risks

Among the 332 self-medicating participants, the mean knowledge index score was 3.12 (SD = 1.62) of 5. Awareness was highest for the two most widely publicized risks: 71.1% (*n* = 236) correctly identified that excessive or prolonged paracetamol use may cause severe liver damage, and 70.2% (*n* = 233) recognized that NSAIDs may cause gastrointestinal irritation and damage.

Awareness was lower in three areas. Fewer than half (47.3%, *n* = 157) correctly identified that paracetamol can provoke allergic reactions, and 56.0% (*n* = 186) correctly identified that NSAIDs are not generally safe for individuals with kidney disease. A further 32.5% failed to reject the misconception that paracetamol is non-toxic at high doses, with 18.4% (*n* = 61) endorsing it as true and 14.2% (*n* = 47) answering “Not sure.” Uncertainty was common across all items, with between 14.2% and 29.8% of participants selecting “Not sure”, highest for paracetamol-related allergic reactions (29.8%, *n* = 99) and lowest for paracetamol toxicity at high doses (14.2%, *n* = 47). Item-level responses are presented in Table 4.

### 3.5. Association Between Lifetime Self-Medication and Sociodemographic Characteristics

Chi-square analysis revealed no statistically significant associations between self-reported self-medication and participants’ sex, age group, or educational level (*p* > 0.05). Although females reported a slightly higher proportion of self-medication than males (85.1% vs. 79.0%), the difference was not statistically significant (χ^2^ = 1.99, *p* = 0.158).

Similarly, self-reported self-medication did not differ significantly across age groups (χ^2^ = 4.57, *p* = 0.335) or educational levels (χ^2^ = 1.34, *p* = 0.246). Self-medication was therefore widely reported across the demographic groups included in the study at the bivariate level (Table 5), although female sex was associated with self-medication after adjustment for age and education, in a model that was only marginally significant (Section 3.7).

### 3.6. Analgesic Safety Knowledge by Sex and Age Group

**Figure 2:**
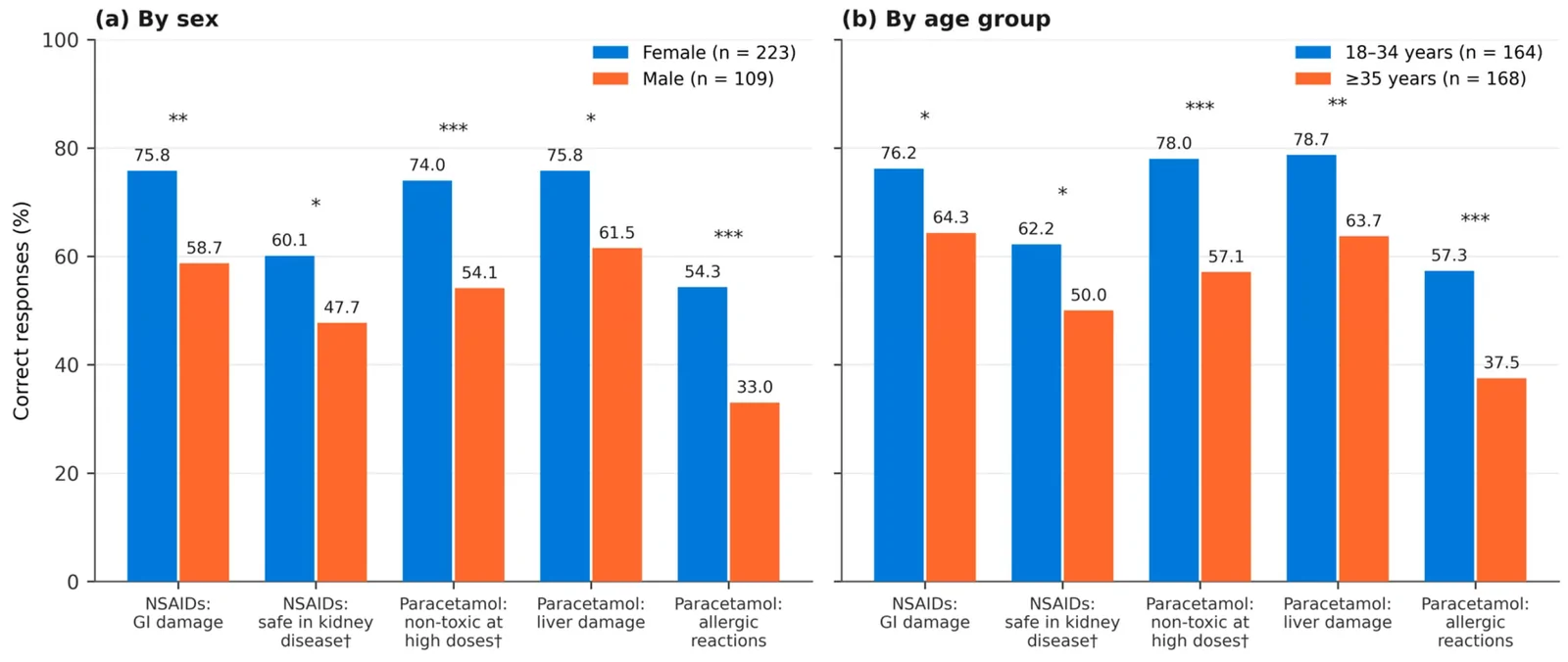
Correct responses to the five analgesic safety knowledge items among self-medicating participants (*n* = 332), by sex ((**a**) female *n* = 223, male *n* = 109) and age group ((**b**) 18–34 years *n* = 164, ≥35 years *n* = 168). † Correct response is “False.” Significance from chi-square tests: *p* < 0.05 (*), *p* < 0.01 (**), *p* < 0.001 (***). Values are percentages answering correctly; “Not sure” responses were scored as incorrect.

Among the 332 self-medicating participants, female participants gave correct responses more often than males on all five knowledge items: NSAID-related gastrointestinal damage (χ^2^ = 9.39, *p* = 0.002), NSAID-related renal risk (χ^2^ = 4.07, *p* = 0.044), paracetamol toxicity at high doses (χ^2^ = 12.27, *p* < 0.001), paracetamol-induced liver damage (χ^2^ = 6.62, *p* = 0.010), and paracetamol-associated allergic reactions (χ^2^ = 12.40, *p* < 0.001). Effect sizes were small (Cramér’s V = 0.11–0.19). Females also had higher knowledge index scores than males (median 4, IQR 2–5 vs. 2, IQR 1–4; U = 15,727, *p* < 0.001) (Table 6; Figure 2a).

As an exploratory analysis, participants were grouped into younger (18–34 years) and older (≥35 years) categories. Younger participants gave correct responses more often on all five items, with the largest differences for paracetamol toxicity at high doses (χ^2^ = 15.59, *p* < 0.001) and paracetamol-associated allergic reactions (χ^2^ = 12.29, *p* < 0.001), and had higher knowledge index scores (median 4, IQR 2–5 vs. 3, IQR 1–4; U = 17,882, *p* < 0.001) (Table 7; Figure 2b). Correct responses on the renal-caution item ranged from 47.7% among males to 62.2% among participants aged 18–34 years.

After Holm–Bonferroni correction across the ten item-level comparisons, seven remained statistically significant. The differences on the renal-risk item by sex and by age group, and on the gastrointestinal item by age group, did not (adjusted *p* = 0.072 for each). The differences in overall knowledge index scores by sex and by age group remained significant (*p* < 0.001). Knowledge index scores did not differ by educational level (median 3, IQR 2–4 for university and above vs. 4, IQR 2–5 for below university; U = 6180, *p* = 0.386).

### 3.7. Psychometric Properties and Multivariable Analyses

Post hoc evaluation of the five-item analgesic safety knowledge index indicated acceptable internal consistency (Cronbach’s α = 0.713; standardized α = 0.715; split-half reliability = 0.740). Principal component analysis supported a predominantly unidimensional structure, with a single component explaining 46.8% of the total variance; a sensitivity analysis based on tetrachoric correlations gave a similar result (first eigenvalue 3.09, 61.9% of variance; second eigenvalue 0.76). Detailed psychometric findings, including item discrimination, inter-item correlations, and factor loadings, are reported in Supplementary Material S4.

**Figure 3:**
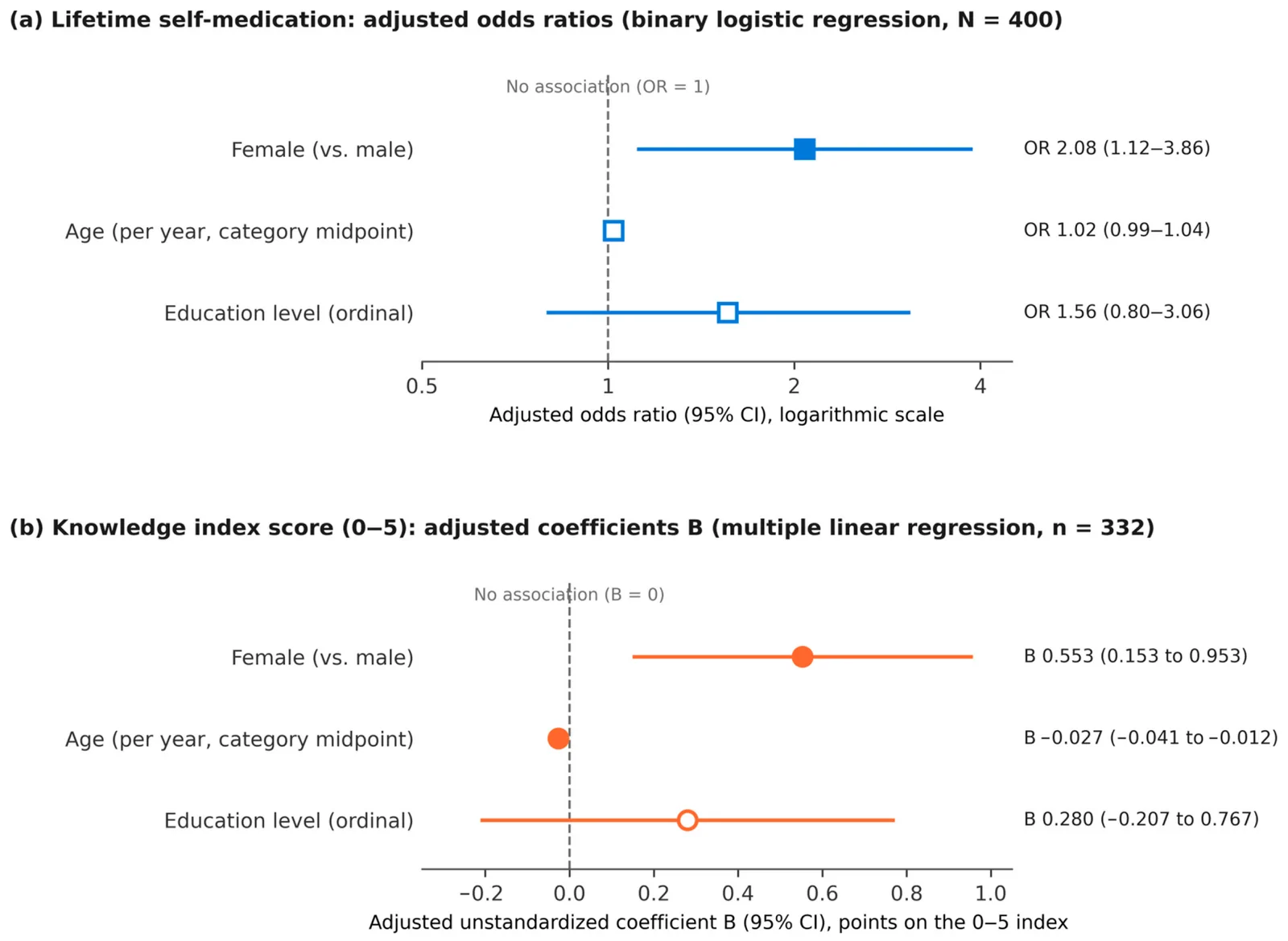
Adjusted associations between sociodemographic characteristics and (**a**) lifetime self-reported analgesic self-medication and (**b**) analgesic safety knowledge score. Panel (**a**) shows adjusted odds ratios from the binary logistic regression model (*n* = 400), plotted on a logarithmic scale with the dashed line at OR = 1. Panel (**b**) shows adjusted unstandardized coefficients (B) from the multiple linear regression model (*n* = 332), expressed in points on the 0-5 knowledge index, with the dashed line at B = 0. The two panels use different measures and scales and should not be compared directly. Bars are 95% confidence intervals; filled markers indicate *p* < 0.05. Age was entered per year using category midpoints. Full model output is given in Table 8.

Exploratory multivariable models were fitted to examine sociodemographic factors associated with self-medication and with analgesic safety knowledge, with sex, age, and educational level entered simultaneously. Table 8 presents full model output, and Figure 3 shows adjusted estimates for the two primary outcomes.

In the logistic model predicting self-medication (Model 1), female sex was the only covariate significantly associated with self-medication (OR = 2.08, 95% CI 1.12–3.86, *p* = 0.020); age (OR = 1.02, 95% CI 0.99–1.04) and educational level (OR = 1.56, 95% CI 0.80–3.06) were not significant. The overall model was only marginally significant (χ^2^(3) = 7.83, *p* = 0.050) and explained a small proportion of variance (Nagelkerke R^2^ = 0.032), indicating that sociodemographic characteristics accounted for little of the variation in self-medication in this sample. The association with female sex should therefore be interpreted with caution and not as strong evidence of an independent predictor.

The association between female sex and self-medication was not significant in the unadjusted analysis (OR = 1.52, 95% CI 0.89–2.59; χ^2^ = 1.99, *p* = 0.158) but was significant after adjustment (OR = 2.08, 95% CI 1.12–3.86) (Table 8). To examine this change, age and education were added to the model separately (Supplementary Material S6). Adjustment for age alone increased the OR for female sex to 2.04 (95% CI 1.10–3.76), whereas adjustment for education alone increased it only to 1.69 (95% CI 0.97–2.92), indicating that the change was attributable mainly to age. Age was strongly related to sex in this sample: 50.0% of the women were aged 18–24 years, compared with 9.4% of the men. Among participants of the same sex, older age showed a weak, non-significant tendency towards more frequent self-medication (OR 1.02 per year of category midpoint, 95% CI 0.999–1.048, *p* = 0.059). Because women were younger, this pattern would conceal a sex difference in the unadjusted comparison, which is consistent with negative confounding by age. Both estimates are reported because they answer different questions: the unadjusted estimate describes the observed difference between women and men in this sample, whereas the adjusted estimate describes the difference among participants of similar age and education. Given that the overall model was only marginally significant, neither estimate should be over-interpreted.

In the linear model predicting knowledge index scores (Model 2), older age was associated with lower scores (B = −0.027 per year of category midpoint, 95% CI −0.041 to −0.012, β = −0.218, *p* < 0.001). Because age was collected in categories, this coefficient approximates the trend across age groups rather than the effect of each additional year. Using the original categories, mean knowledge scores declined from 3.65 among participants aged 18–24 years to 3.20, 2.90, 2.73 and 2.21 among those aged 25–34, 35–44, 45–54 and ≥55 years, respectively. In the adjusted categorical model, scores were lower than in the 18–24-year group by 0.68 points (95% CI 0.22–1.15) at 35–44 years, 0.77 points (95% CI 0.23–1.31) at 45–54 years and 1.13 points (95% CI 0.42–1.84) at ≥55 years (Supplementary Material S6). Female sex was associated with modestly higher scores (B = 0.55, 95% CI 0.15–0.95, β = 0.160, *p* = 0.007). Educational level was not significant (B = 0.28, 95% CI −0.21 to 0.77, *p* = 0.259). The model explained 9.7% of the variance in knowledge scores (R^2^ = 0.097, adjusted R^2^ = 0.088). A sex × age interaction term was not significant (ΔR^2^ = 0.001, *p* = 0.485), indicating that the association between age and knowledge was similar in both sexes.

When knowledge was dichotomized at the adequacy threshold (≥4 of 5 correct; Model 3), both female sex (OR = 2.21, 95% CI 1.29–3.77, *p* = 0.004) and older age (OR = 0.968 per year of category midpoint, 95% CI 0.949–0.988, *p* = 0.002) were associated with adequate knowledge. In the categorical model, the odds of adequate knowledge were lower than in the 18–24-year group at 35–44 years (OR 0.38, 95% CI 0.20–0.71), 45–54 years (OR 0.45, 95% CI 0.22–0.92) and ≥55 years (OR 0.27, 95% CI 0.10–0.76) (Supplementary Material S6). Educational level was not significant (OR = 1.61, 95% CI 0.84–3.06, *p* = 0.148). Models 2 and 3 therefore converged, with both female sex and younger age associated with higher safety knowledge.

Estimates were stable across alternative codings of age (Supplementary Material S6). In the self-medication model, the adjusted OR for female sex ranged from 1.94 to 2.08 (all *p* < 0.05). In the knowledge models, the coefficient for female sex ranged from 0.53 to 0.65 in Model 2 and the adjusted OR from 2.17 to 2.42 in Model 3 (all *p* < 0.05), and age remained inversely associated with knowledge under every coding. Categorical coding did not improve model fit over the midpoint term in any model (Model 1: χ^2^(3) = 4.05, *p* = 0.257; Model 2: F(3, 325) = 0.64, *p* = 0.591; Model 3: χ^2^(3) = 2.82, *p* = 0.420). Replacing the ordinal education variable with a binary variable (university vs. school education) did not materially change any estimate. Education remained non-significant in all three models (Model 1: OR 1.54, 95% CI 0.76–3.15; Model 2: B = 0.25, 95% CI −0.25 to 0.76; Model 3: OR 1.54, 95% CI 0.79–3.00), and the estimates for female sex and age changed by 0.04 or less. When adequate knowledge was redefined using cut-offs of ≥3/5 or 5/5, older age remained significantly associated with lower odds of adequate knowledge at both thresholds (OR 0.96, 95% CI 0.94–0.98, and OR 0.95, 95% CI 0.93–0.98, respectively), whereas female sex was associated with adequate knowledge at the ≥3/5 threshold (OR 1.93, 95% CI 1.13–3.31) but not at 5/5 (OR 1.24, 95% CI 0.66–2.34). Internal consistency of the knowledge index was similar in the English and Arabic versions (Cronbach’s α = 0.707 and 0.695, respectively). Participants who completed the Arabic version had lower knowledge scores (median 3, IQR 1–4 vs. 4, IQR 2–5; *p* < 0.001), and this difference persisted after adjustment for sex, age and education (B = −0.59, 95% CI −1.03 to −0.15). Adding questionnaire language to the models did not materially change the estimates for female sex (Model 1: OR 1.95, 95% CI 1.04–3.67; Model 2: B = 0.64, 95% CI 0.24–1.04; Model 3: OR 2.29, 95% CI 1.33–3.94) or age, which remained significantly associated with knowledge (Supplementary Material S6).

## 4. Discussion

In this UAE-based sample, 83.0% of participants reported having ever used analgesics without professional medical consultation. Because this is a lifetime measure obtained from a convenience sample, it indicates that analgesic self-medication was a familiar practice among respondents rather than providing a population prevalence estimate, and it is not directly comparable with studies reporting 12-month or point prevalence. The finding is nonetheless consistent with international reports that analgesic self-medication is a common component of self-care where over-the-counter medications are easily accessible [1,2]. Comparison with earlier UAE studies requires attention to differences in design. AlKhalidi et al. [28] surveyed 400 community adults face to face in several emirates and reported self-medication in the previous 12 months by 78% of participants, most often with painkillers; more than half were unaware of drug–drug and food–drug interactions. Alzoubi et al. [7] reported that about 60% of university students used over-the-counter medicines without a prescription. The present figure of 83.0% is in line with these reports but cannot be compared with them directly, because it refers to lifetime rather than 12-month use, was obtained through an online rather than a face-to-face survey, was restricted to analgesics, and comes from a sample with a different age and educational profile. The present study adds item-level knowledge results, the distinction between incorrect and uncertain responses, and the actions taken when self-medication fails, which were not reported in these earlier UAE studies.

Paracetamol was the most frequently used analgesic (93.7%), followed by ibuprofen (58.7%) and diclofenac (39.2%), consistent with regional reports in which paracetamol is perceived as a safe and familiar first-line option and NSAIDs are valued for rapid relief [29,30]. A similar order was reported among UAE university students, of whom 80.9% used paracetamol and 35.4% ibuprofen [31]. Although this pattern of use may be consistent with reasonable therapeutic choices, NSAID use without adequate safety knowledge may carry potential risks, because NSAIDs are associated with gastrointestinal, renal and cardiovascular complications, particularly with repeated or excessive use [24], although adverse outcomes were not assessed in this study. Underestimation of long-term NSAID risks has been reported in other populations [32,33], but whether it applies here could not be determined, because the frequency, dose and duration of NSAID use were not recorded.

Motivations for self-medication were primarily pragmatic, with more than half of the participants citing previous positive experience and convenience. These reasons are consistent with findings among university populations in the UAE, Jordan, and Saudi Arabia [7,31,34], and perceiving the illness as not serious was also the most common reason in the UAE community study [28]. However, the role of financial difficulty or lack of insurance, reported by 11.4% of participants, appears less pronounced in this study than in lower-income settings, where limited healthcare access and out-of-pocket expenses are associated with higher rates of self-medication [35,36]. In the UAE, where public and employer-funded healthcare is more widespread, financial constraints may be less influential. Convenience and the perception of symptoms as minor were the most commonly reported reasons for self-care in this sample.

No statistically significant bivariate associations were identified between self-medication and sex (χ^2^ = 1.99, *p* = 0.158), age group (χ^2^ = 4.57, *p* = 0.335), or educational level (χ^2^ = 1.34, *p* = 0.246). In the exploratory multivariable model, female sex was associated with self-medication (aOR 2.08, 95% CI 1.12–3.86), a change from the unadjusted estimate that is consistent with negative confounding by age (Section 3.7), but sociodemographic characteristics together explained little of the variation (Nagelkerke R^2^ = 0.032). This association should be interpreted cautiously, because the overall model was only marginally significant (*p* = 0.050) and the estimate emerged only after adjustment. Because participants were not randomly sampled, this should be read as limited subgroup variation within the sample rather than as evidence about the distribution of self-medication in the UAE population. The sample contained too few participants without university education (*n* = 61 combined) to assess educational gradients meaningfully.

While most participants reported that they would seek professional consultation when self-medication failed, a minority reported practices with potential safety implications. In particular, 14.8% reported increasing the dose of the same medicine without professional guidance. Unsupervised dose escalation is a recognized risk factor for toxicity with analgesics that have defined maximum daily doses, particularly paracetamol [25], although this study did not record the extent of dose escalation or measure adverse outcomes. Similar practices have been reported in Nigeria and Egypt [37,38]. These responses suggest a trial-and-error approach to symptom management. Whether they resulted in harm could not be assessed, because cumulative doses, duration of use, concomitant medications and clinical outcomes were not measured.

In the five items assessed, knowledge of analgesic safety in this sample was uneven rather than uniformly limited, and the distribution of what was and was not recognized is more informative than the composite score alone. Awareness of the two most widely communicated risks was relatively high: over 70% of participants identified both paracetamol-related hepatotoxicity and NSAID-related gastrointestinal effects. Recognition was lower for risks that appear to receive less public attention. Fewer than half (47.3%) were aware that paracetamol can provoke allergic reactions, only 56.0% identified that NSAIDs require caution in individuals with kidney disease, and approximately one-third (32.5%) did not recognize that paracetamol carries dose-dependent toxicity. Selective recognition of analgesic risks has also been reported in community samples in Indonesia, Thailand, and Cyprus, where recognition of paracetamol-related risks exceeded recognition of NSAID-related risks and knowledge was higher among those who had received side-effect information [39,40,41], which suggests that these gaps may be related to how analgesic risk information is communicated rather than to characteristics of this sample, although this explanation could not be tested here. On the renal item, uncertainty was more common than misconception (26.8% “Not sure” vs. 17.2% incorrect), a distinction that may matter for education (Section Implications for Health Education and Prevention). Because the knowledge index was a brief, study-specific exploratory measure covering selected high-priority concepts, the item-level findings are best read as indicators of specific gaps in understanding rather than as an overall measure of analgesic safety literacy.

Sex and age differences in knowledge were observed, with females and younger participants scoring higher. Both associations were consistent across the exploratory multivariable analyses: female sex and younger age were associated with higher knowledge whether knowledge was modelled as a continuous score or dichotomized at ≥4/5, and under every coding of age. The age association was also present at the alternative thresholds of ≥3/5 and 5/5, whereas the sex association was not significant at the strictest 5/5 threshold. Because age was measured in categories, the per-year estimates should be read as approximate trends across age groups rather than precise effects of each additional year; the categorical analysis showed the same pattern (Supplementary Material S6). The sex difference was modest, amounting to about half a point on the five-point index (B = 0.55), and should not be over-interpreted. Effect sizes were small (Cramér’s V 0.11–0.22), and three of the ten item-level differences did not remain significant after correction for multiple comparisons, so individual item differences should be interpreted cautiously. The study did not assess health literacy, information-seeking behavior, or media exposure, so these differences should be interpreted without causal assumptions. Healthcare background, which is strongly associated with medication knowledge, was not recorded and may account for part of these differences.

As clinical context, older adults are generally more vulnerable to NSAID-associated renal and gastrointestinal complications [24,33]; however, this study did not assess comorbidities, renal function, gastrointestinal history, concomitant medications or NSAID exposure, so it cannot show that older participants were at higher clinical risk. Because the age gradient was similar in both sexes, this observation, if confirmed, may be more relevant to the content and reach of educational messaging than to demographic targeting.

This study has several limitations. The most important is the convenience-based online sampling. The sample was disproportionately young (50.5% aged 18–34 years), highly educated (84.8% with university education) and female (65.5%), and therefore does not reflect the demographic composition of the UAE population. Eligibility was self-reported, and residency status and current prescribed analgesic therapy could not be independently verified, so some ineligible respondents may have been included. Online recruitment is also likely to have excluded individuals with limited digital access. Because the invitation advertised a downloadable educational leaflet on pain medications, the survey may also have preferentially attracted people already interested in medication safety; this is a potential source of selection bias that would tend to raise knowledge scores, so the knowledge gaps reported here may underestimate those in the wider population. Occupation and field of study, including whether participants had a healthcare background, were not recorded. Because the survey was distributed through the social networks of medical students, and some free-text responses indicated healthcare employment, participants with medical or pharmacy training may have been over-represented, which could have raised both self-medication confidence and knowledge scores relative to the general population. Because knowledge scores were higher among younger respondents, the knowledge levels observed here may overestimate those of the wider population. The findings therefore describe this sample and should not be generalized to the UAE population as a whole. In addition, the self-medication question had no defined recall period, so the 83.0% figure reflects lifetime ever-use and cannot be compared directly with period-prevalence estimates; together with the sampling approach, this means it should not be read as a population prevalence. The sample size was calculated for a single proportion rather than for the regression models, which were therefore exploratory; although the number of events per predictor was adequate, the models were not powered a priori to detect specific effect sizes. Age was collected in categories and modelled through category midpoints, which assumes a uniform age distribution within each band and may attenuate the age coefficient, although the estimates were materially unchanged when age was modelled as ordinal, categorical or binary (Supplementary Material S6). Minor differences between the English and Arabic response options are described in Section 2.3. Knowledge items were administered only to those reporting self-medication, so knowledge among the 68 non-self-medicating participants was not assessed. In addition, the reported *p* values were not adjusted for multiple comparisons, and three of the ten item-level differences by sex and age group did not remain significant after Holm–Bonferroni correction. Sex was assessed as a binary variable, so broader sex-related factors such as social roles and access to health information were not captured. Concomitant medication use and polytherapy were not assessed, limiting evaluation of potential drug interactions. More generally, the study measured knowledge and self-reported practices only; it did not measure actual adverse drug events, overdose severity, cumulative doses, duration of use, drug interactions or clinical outcomes, so it cannot show whether the knowledge gaps or practices observed lead to harm. The knowledge index was a brief, study-specific exploratory measure whose validity was not formally established beyond expert review and internal consistency; it was piloted in only eight participants to check clarity, and it omitted maximum daily dose, duplicate ingredient exposure across combination products, drug–drug interactions, contraindications and high-risk clinical populations. Equivalence between the English and Arabic versions was not formally tested, and the back-translation process was not documented. Participants who completed the Arabic version had lower knowledge scores than those who completed the English version, even after adjustment for sex, age and education; this may reflect differences between the two groups of respondents, differences in how the items functioned in the two languages, or both, and cannot be resolved with the present data. The ≥4/5 threshold for adequate knowledge is an exploratory operational categorization based on a conventional 80% cut-off rather than a clinically validated standard. Future studies should pair a standardized definition with a specified recall window, record exact age in years, occupation and healthcare training, incorporate broader clinical and healthcare-access variables, and use probability-based sampling where feasible.

### Implications for Health Education and Prevention

The following implications are offered as hypotheses for future intervention research rather than as established recommendations, because the cross-sectional design identifies associations and knowledge gaps but cannot establish causal pathways or the effectiveness of any intervention. Three features of these findings may be relevant to how medication-safety education is designed.

First, on the five items assessed, safety knowledge was selective rather than uniformly limited. Recognition of hepatotoxicity and gastrointestinal risk exceeded 70%, whereas fewer than half recognized allergic potential and only 56.0% identified renal caution. General messaging that analgesics carry risks may therefore add little for this group, and educational content addressing the domains less often recognized in this sample could be developed and evaluated.

Second, uncertainty was more common than misconception on the renal-risk item: 26.8% of participants answered “Not sure” and 17.2% answered incorrectly. These may represent educationally distinct problems: uncertainty may respond to straightforward information provision, whereas established misconceptions may require active correction. Distinguishing the two could help programs direct their effort more efficiently.

Third, some of the behaviors observed may indicate gaps in procedural rather than declarative knowledge. Participants who increased the dose (14.8%) or switched medication (29.8%) when initial relief was not achieved may not lack awareness that analgesics carry risks; they may instead lack guidance on the appropriate action when a standard dose fails. Content framed only as risk awareness may not address this, and explicit guidance on what to do and when to seek advice is a possible content area for future evaluation.

These observations also bear on delivery. Sociodemographic characteristics explained little of the variation in self-medication (Nagelkerke R^2^ = 0.032), which is consistent with the practice being widespread across groups in this sample rather than confined to a definable at-risk population. If this is confirmed in representative samples, approaches delivered at the point of access may be worth exploring alongside approaches targeted at specific groups. Nearly half of participants (47.6%) cited ease of accessibility as a factor in analgesic selection, and community pharmacies, as a common point of access to over-the-counter analgesics, may be a practical setting for brief structured counselling. Pharmacist-led interventions have improved consumer knowledge and reduced inappropriate analgesic use in other settings [42,43]; however, the present study did not evaluate any intervention, and whether pharmacist counselling is effective in the UAE, or more effective than other approaches, is not known and would require evaluation. This is relevant given the UAE’s updated national pharmaceutical regulatory framework, which reinforces oversight of medical products and pharmacy practice [44].

Although they were not examined in this study, system-level measures such as clearer labelling of analgesic warnings and restrictions on pack size for medications with well-defined toxicity thresholds could complement counselling at the point of sale; pack-size restrictions have been implemented in several jurisdictions, including Australia’s 2025 reduction in non-pharmacy paracetamol pack sizes [45].

## 5. Conclusions

In this online convenience sample, lifetime analgesic self-medication was reported by most respondents, most commonly with paracetamol and ibuprofen, and sociodemographic characteristics explained little of the variation in this practice. Among self-medicating respondents, safety knowledge, as measured by a brief study-specific index, was uneven rather than uniformly limited: hepatic and gastrointestinal risks were widely recognized, whereas renal caution, allergic potential, and dose-dependent toxicity were less often recognized. A minority reported dose escalation or medication switching when initial relief was not achieved.

Because the study was cross-sectional and its multivariable analyses were exploratory, these findings suggest two hypotheses, rather than recommendations, for prevention practice. First, because the knowledge gaps identified were specific to particular items rather than general, educational content that addresses the less-recognized domains may be more useful than content that reinforces risks that respondents already recognized. Second, because self-medication was reported across all sociodemographic groups in this sample, delivery at the point of access, for example through community pharmacies, may be worth evaluating. Both hypotheses should be tested in intervention studies.

Because the findings derive from a convenience online sample that over-represented younger, university-educated and female adults, they describe the participants studied and should be confirmed in population-based studies before being generalized to the UAE population. They nonetheless identify specific, item-level gaps that may help inform the design of medication-safety education, and the accompanying brochure offers an intervention component for prospective evaluation.

## Figures and Tables

**Table 1:** Sociodemographic Profile of Study Participants (*n* = 400).

Variable	*n* (%)
Sex	
Female	262 (65.5)
Male	138 (34.5)
Age group (years)	
18–24	144 (36.0)
25–34	58 (14.5)
35–44	103 (25.8)
45–54	60 (15.0)
≥55	35 (8.8)
Highest education level	
Primary school	2 (0.5)
Secondary school	59 (14.8)
University and above	339 (84.8)

**Table 2:** Lifetime Self-Reported Self-Medication, Analgesics Used, and Primary Symptoms.

Variable	*n* (%)
Lifetime self-medication (*n* = 400)	
Yes	332 (83.0)
No	68 (17.0)
Analgesics used (*n* = 332)	
Paracetamol	311 (93.7)
Ibuprofen	195 (58.7)
Diclofenac	130 (39.2)
Mefenamic acid	59 (17.8)
Aspirin	51 (15.4)
Primary symptoms (*n* = 332)	
Headache	256 (77.1)
Fever	177 (53.3)
Menstrual pain	118 (35.5)
Muscular pain	105 (31.6)
Dental pain	95 (28.6)
Runny nose	85 (25.6)
Joint pain	84 (25.3)
Cough	76 (22.9)
Diarrhea	30 (9.0)
Post-surgical pain	10 (3.0)

Values are *n* (%). Multiple responses were permitted for analgesics used and primary symptoms; percentages therefore exceed 100%. The questionnaire listed common brand names alongside the generic names (Panadol, Brufen, Voltaren and Ponstan).

**Table 3:** Motivations, Influencing Factors, and Actions Taken When Self-Medication Fails (*n* = 332).

Variable	*n* (%)
Motivations	
Previous positive experiences	193 (58.1)
Convenience	192 (57.8)
Mild illness	190 (57.2)
Cost effectiveness	114 (34.3)
Lack of time	110 (33.1)
Lack of health insurance	38 (11.4)
Factors influencing choice	
Previous effectiveness	259 (78.0)
Previous doctor’s recommendation	162 (48.8)
Ease of accessibility	158 (47.6)
Affordability	90 (27.1)
Concerns about side effects	68 (20.5)
Actions when self-medication fails	
Seeking medical consultation	200 (60.2)
Waiting for symptoms to resolve	103 (31.0)
Using a different medication	99 (29.8)
Alternative medicine/home remedies	72 (21.7)
Double dosing	49 (14.8)
Seeking friends’ or family’s advice	44 (13.3)

Values are *n* (%). Multiple responses were permitted; percentages therefore exceed 100%.

**Table 4:** Knowledge of Analgesic Safety Risks Among Self-Medicating Participants (*n* = 332).

Knowledge Statement	True, *n* (%)	False, *n* (%)	Not Sure, *n* (%)	Correct, %
NSAIDs can cause irritation and damage to the lining of the stomach and intestines.	233 (70.2)	26 (7.8)	73 (22.0)	70.2
NSAIDs are generally safe for individuals with kidney problems. ^ab^	57 (17.2)	186 (56.0)	89 (26.8)	56.0
Paracetamol (acetaminophen) has no risk of toxicity, even at high doses ^a^	61 (18.4)	224 (67.5)	47 (14.2)	67.5
Excessive or prolonged use of paracetamol can lead to severe liver damage.	236 (71.1)	32 (9.6)	64 (19.3)	71.1
In rare cases, paracetamol can cause allergic reactions like skin rash, swelling, etc.	157 (47.3)	76 (22.9)	99 (29.8)	47.3
Knowledge index score (0–5), mean (SD)				3.12 (1.62)

Statements are shown exactly as worded in the English version of the questionnaire (Supplementary Material S3). NSAIDs, non-steroidal anti-inflammatory drugs. ^a^ Correct answer is ‘False’. ‘Not sure’ responses were scored as incorrect. The knowledge index score is the number of correct responses across the five items (range 0–5). ^b^ In the Arabic version, this statement was worded negatively (“NSAIDs are generally not safe for individuals with kidney problems”); Arabic-version responses are shown as their English-version equivalents (Section 2.3).

**Table 5:** Lifetime Self-Reported Self-Medication by Sociodemographic Characteristics (*n* = 400).

Characteristic	Yes, *n* (%)	No, *n* (%)	χ^2^	*p*
Sex			1.99 ^a^	0.158
Female	223 (85.1)	39 (14.9)		
Male	109 (79.0)	29 (21.0)		
Age group (years)			4.57	0.335
18–24	119 (82.6)	25 (17.4)		
25–34	45 (77.6)	13 (22.4)		
35–44	84 (81.6)	19 (18.4)		
45–54	55 (91.7)	5 (8.3)		
≥55	29 (82.9)	6 (17.1)		
Education			1.34 ^a^	0.246
Below university	47 (77.0)	14 (23.0)		
University and above	285 (84.1)	54 (15.9)		

Values are *n* (row %). All expected cell counts exceeded five. ^a^ Continuity-corrected (Yates) chi-square for 2 × 2 comparisons. No comparison was significant at *p* < 0.05; adjusted associations are reported in Section 3.7.

**Table 6:** Knowledge of Analgesic Safety by Sex Among Self-Medicating Participants (*n* = 332).

Item	Female (*n* = 223), % Correct	Male (*n* = 109), % Correct	χ^2^	*p*	V
NSAIDs cause GI damage	75.8	58.7	9.39	0.002	0.17
NSAIDs safe for kidneys ^a^	60.1	47.7	4.07	0.044	0.11
Paracetamol non-toxic at high doses ^a^	74.0	54.1	12.27	<0.001	0.19
Paracetamol causes liver damage	75.8	61.5	6.62	0.010	0.14
Paracetamol allergic reactions	54.3	33.0	12.40	<0.001	0.19
Knowledge index (0–5), median (IQR)	4 (2–5)	2 (1–4)	U = 15,727 ^b^	<0.001	

Item labels are abbreviated; the full wording of each statement is given in Table 4. GI, gastrointestinal; IQR, interquartile range; V, Cramér’s V. “Not sure” responses were scored as incorrect. Item comparisons used continuity-corrected chi-square tests; *p* values are unadjusted (Holm–Bonferroni-adjusted results are reported in Section 3.6). ^a^ Correct answer is “False”. ^b^ Mann–Whitney U test. Arabic-version responses to the renal item were scored as described in Section 2.3.

**Table 7:** Knowledge of Analgesic Safety by Age Group Among Self-Medicating Participants (*n* = 332).

Item	18–34 Years (*n* = 164), % Correct	≥35 Years (*n* = 168), % Correct	χ^2^	*p*	V
NSAIDs cause GI damage	76.2	64.3	5.09	0.024	0.12
NSAIDs safe for kidneys ^a^	62.2	50.0	4.53	0.033	0.12
Paracetamol non-toxic at high doses ^a^	78.0	57.1	15.59	<0.001	0.22
Paracetamol causes liver damage	78.7	63.7	8.33	0.004	0.16
Paracetamol allergic reactions	57.3	37.5	12.29	<0.001	0.19
Knowledge index (0–5), median (IQR)	4 (2–5)	3 (1–4)	U = 17,882 ^b^	<0.001	

Item labels are abbreviated; the full wording of each statement is given in Table 4. GI, gastrointestinal; IQR, interquartile range; V, Cramér’s V. “Not sure” responses were scored as incorrect. Item comparisons used continuity-corrected chi-square tests; *p* values are unadjusted (Holm–Bonferroni-adjusted results are reported in Section 3.6). This dichotomous age grouping was used for an exploratory analysis and should be interpreted alongside the original age-category analysis and the multivariable models. ^a^ Correct answer is “False”. ^b^ Mann–Whitney U test. Arabic-version responses to the renal item were scored as described in Section 2.3.

**Table 8:** Unadjusted and multivariable models of lifetime self-medication and analgesic safety knowledge.

(A) Binary logistic regression models (Models 1 and 3)							
Predictor		Unadjusted OR (95% CI)	*p*	Adjusted B	SE	Adjusted OR (95% CI)	*p*
Model 1. Lifetime self-medication (*n* = 400) ^a^							
Female sex		1.52 (0.89–2.59)	0.122	0.733	0.315	2.08 (1.12–3.86)	0.02
Age (per year, category midpoint)		1.01 (0.99–1.03)	0.377	0.018	0.013	1.02 (0.99–1.04)	0.139
Education level		1.61 (0.86–2.99)	0.134	0.446	0.343	1.56 (0.80–3.06)	0.193
Model 3. Adequate knowledge, ≥4/5 correct (*n* = 332) ^b^							
Female sex		2.97 (1.83–4.82)	<0.001	0.791	0.273	2.21 (1.29–3.77)	0.004
Age (per year, category midpoint)		0.96 (0.94–0.98)	<0.001	−0.032	0.01	0.97 (0.95–0.99)	0.002
Education level		0.95 (0.52–1.72)	0.86	0.475	0.328	1.61 (0.84–3.06)	0.148
(B) Multiple linear regression model (Model 2)							
Predictor	Unadjusted B (95% CI)	*p*	Adjusted B	SE	95% CI	β	*p*
Model 2. Knowledge index score, 0–5 (*n* = 332) ^c^							
Female sex	0.849 (0.486 to 1.211)	<0.001	0.553	0.203	0.153 to 0.953	0.16	0.007
Age (per year, category midpoint)	−0.033 (−0.046 to −0.021)	<0.001	−0.027	0.007	−0.041 to −0.012	−0.218	<0.001
Education level	−0.144 (−0.632 to 0.343)	0.561	0.28	0.248	−0.207 to 0.767	0.062	0.259

OR, odds ratio; B, unstandardized regression coefficient (log-odds in panel A); SE, standard error of B; CI, confidence interval; β, standardized regression coefficient. Unadjusted estimates are from single-predictor models; adjusted estimates are from models including all three predictors. Sex coded 0 = male, 1 = female; education entered as ordinal (1 = primary, 2 = secondary, 3 = university or above); age entered using category midpoints. The difference between unadjusted and adjusted ORs for female sex in Model 1 is consistent with negative confounding by age (Section 3.7; Supplementary Material S6). Sensitivity analyses with alternative coding of age and education and with questionnaire language are reported in Section 3.7 and Supplementary Material S6. ^a^ Model χ^2^(3) = 7.83, *p* = 0.050; Nagelkerke R^2^ = 0.032; Hosmer–Lemeshow χ^2^(6) = 4.08, *p* = 0.665. ^b^ Model χ^2^(3) = 31.16, *p* < 0.001; Nagelkerke R^2^ = 0.119. ^c^ F(3, 328) = 11.68, *p* < 0.001; R^2^ = 0.097; adjusted R^2^ = 0.088.

## Data Availability

The data presented in this study are available on request from the corresponding authors due to ethical and privacy restrictions. The dataset contains information obtained from human participants, and the data cannot be made publicly available to protect participant confidentiality and privacy. Requests for access to the data will be considered by the corresponding author subject to applicable ethical and institutional requirements.

## Supplementary Materials

The following supporting information can be downloaded at: https://www.mdpi.com/article/10.3390/healthcare14213655/s1, S1: STROBE checklist. S2: CHERRIES checklist. S3: English and Arabic versions of the self-administered survey questionnaire. S4: Educational brochure on analgesic safety, as distributed to participants. S5: Post hoc psychometric evaluation of the study-specific five-item analgesic safety knowledge index. S6: Sensitivity analysis of the multivariable models of lifetime analgesic self-medication (*n* = 400) and safety knowledge (*n* = 332): age coding, confounding by age, and questionnaire language.

## Abbreviations

The following abbreviations are used in this manuscript:

NSAIDs	Non-Steroidal Anti-Inflammatory Drugs
OTC	Over-The-Counter
