# Supplementary material for: Analgesic Self-Medication and Safety Knowledge Among Adults in the United Arab Emirates: A Cross-Sectional Online Survey
Source: Healthcare (Basel). 2026 Oct 28;14(21):3655. doi: 10.3390/healthcare14213655 (PMC13762553; doi:10.3390/healthcare14213655)
Supplement: Supplementary file 1 [file healthcare-14-03655-s001.zip › healthcare-4574820-supplementary.pdf]

Supplementary Material S1

STROBE Statement — Checklist of Items for Reports of Cross-Sectional Studies

Manuscript: Analgesic Self-Medication and Safety Knowledge Among Adults in the United Arab Emirates: A Cross-Sectional Online Survey

No.	Item	Recommendation	Reported in
**Title and Abstract**			
1	**Title and abstract**	(a) Indicate the study's design with a commonly used term in the title or the abstract.	Title; Abstract, Methods
		(b) Provide in the abstract an informative and balanced summary of what was done and what was found.	Abstract
**Introduction**			
2	**Background / rationale**	Explain the scientific background and rationale for the investigation being reported.	Section 1, paragraphs 1–3
3	**Objectives**	State specific objectives, including any prespecified hypotheses.	Section 1, final paragraph
**Methods**			
4	**Study design**	Present key elements of study design early in the paper.	Section 2.1
5	**Setting**	Describe the setting, locations, and relevant dates, including periods of recruitment, exposure, follow-up, and data collection.	Section 2.1; Section 2.2
6	**Participants**	(a) Give the eligibility criteria, and the sources and methods of selection of participants.	Section 2.2
7	**Variables**	Clearly define all outcomes, exposures, predictors, potential confounders, and effect modifiers. Give diagnostic criteria, if applicable.	Section 2.3; Section 2.5
8*	**Data sources / measurement**	For each variable of interest, give sources of data and details of methods of assessment (measurement). Describe comparability of assessment methods if there is more than one group.	Section 2.3; Supplementary Material S3; Supplementary Material S4
9	**Bias**	Describe any efforts to address potential sources of bias.	Section 2.2; Section 2.3; Section 4 (Limitations)
10	**Study size**	Explain how the study size was arrived at.	Section 2.4
11	**Quantitative variables**	Explain how quantitative variables were handled in the analyses. If applicable, describe which groupings were chosen and why.	Section 2.6
12	**Statistical methods**	(a) Describe all statistical methods, including those used to control for confounding.	Section 2.6

No.	Item	Recommendation	Reported in
		(b) Describe any methods used to examine subgroups and interactions.	Section 2.6; Section 3.6; Section 3.7
		(c) Explain how missing data were addressed.	Section 2.6; Section 3.1 (only complete responses were analysed; no missing data)
		(d) If applicable, describe analytical methods taking account of sampling strategy.	Not applicable; convenience sample, unweighted
		(e) Describe any sensitivity analyses.	Section 2.6; Section 3.7; Supplementary Material S6
**Results**			
13*	**Participants**	(a) Report numbers of individuals at each stage of study — e.g. numbers potentially eligible, examined for eligibility, confirmed eligible, included in the study, completing follow-up, and analysed.	Section 2.2; Section 3.1; Figure 1
		(b) Give reasons for non-participation at each stage.	Section 3.1; Figure 1 (see Note 1 below)
		(c) Consider use of a flow diagram.	Figure 1
14*	**Descriptive data**	(a) Give characteristics of study participants and information on exposures and potential confounders.	Section 3.1; Table 1
		(b) Indicate number of participants with missing data for each variable of interest.	Section 3.1 (no missing data among the 400 complete responses)
15*	**Outcome data**	Report numbers of outcome events or summary measures.	Sections 3.2–3.4; Tables 2–4
16	**Main results**	(a) Give unadjusted estimates and, if applicable, confounder-adjusted estimates and their precision (e.g. 95% CI). Make clear which confounders were adjusted for and why they were included.	Sections 3.5–3.7; Tables 5–8; Figures 2 and 3
		(b) Report category boundaries when continuous variables were categorized.	Section 2.6; Table 1; Section 3.6
		(c) If relevant, consider translating estimates of relative risk into absolute risk for a meaningful time period.	Not applicable
17	**Other analyses**	Report other analyses done — e.g. analyses of subgroups and interactions, and sensitivity analyses.	Section 3.6; Section 3.7; Supplementary Materials S4 and S6
**Discussion**			
18	**Key results**	Summarise key results with reference to study objectives.	Section 4, opening paragraphs

No.	Item	Recommendation	Reported in
19	**Limitations**	Discuss limitations of the study, taking into account sources of potential bias or imprecision. Discuss both direction and magnitude of any potential bias.	Section 4 (Limitations)
20	**Interpretation**	Give a cautious overall interpretation of results considering objectives, limitations, multiplicity of analyses, results from similar studies, and other relevant evidence.	Section 4; Section 5
21	**Generalisability**	Discuss the generalisability (external validity) of the study results.	Section 4 (Limitations)
**Other Information**			
22	**Funding**	Give the source of funding and the role of the funders for the present study and, if applicable, for the original study on which the present article is based.	Funding statement

*Give information separately for exposed and unexposed groups where applicable.

Note 1. Because the survey was distributed openly through social media and institutional channels, the number of individuals who saw the invitation without opening the survey link could not be recorded, and reasons for non-participation were not collected. The survey link was accessed 409 times; 401 began the questionnaire and 400 submitted complete responses; one incomplete response was excluded (Section 3.1; Figure 1).

Reference: von Elm, E.; Altman, D.G.; Egger, M.; Pocock, S.J.; Gøtzsche, P.C.; Vandenbroucke, J.P. The Strengthening the Reporting of Observational Studies in Epidemiology (STROBE) statement: guidelines for reporting observational studies. Lancet 2007, 370, 1453–1457. An Explanation and Elaboration article is available at https://www.strobe-statement.org.

Supplementary Material S2

CHERRIES — Checklist for Reporting Results of Internet E-Surveys

Manuscript: Analgesic Self-Medication and Safety Knowledge Gaps Among Adults in the United Arab Emirates: A Cross-Sectional Online Survey

Checklist item	Explanation	Reported in the manuscript / response
**Describe survey design**	Describe target population, sample frame. Is the sample a convenience sample?	Section 2.2. Target population: adults aged ≥18 years residing in the UAE. Individuals receiving prescribed analgesic therapy under medical supervision, those under 18, and temporary visitors were excluded. No formal sampling frame existed; a non-probability convenience sample was recruited online. This limitation is stated in Sections 2.2 and 4.
**IRB approval**	Mention whether the study has been approved by an IRB.	Section 2.7. Approved by the Research and Ethics Committee of Dubai Medical University (Approval Reference No. REC/DMCG/AY23-24/F 3).
**Informed consent**	Describe the informed consent process. Where were participants told the length of the survey, which data were stored and where and for how long, who the investigator was, and the purpose of the study?	Sections 2.1, 2.2 and 2.7. An electronic informed consent page preceded the survey items. It identified the researchers and their institution (Dubai Medical College for Girls), provided a contact email, stated the purpose of the study and the estimated completion time (3–5 minutes), confirmed confidentiality and anonymity, and informed participants of their right to withdraw at any point. Participants indicated consent before proceeding. The consent page did not state a data retention period or storage location.
**Data protection**	If any personal information was collected or stored, describe what mechanisms were used to protect unauthorized access.	Section 2.7. No personally identifying information was collected; all responses were anonymous and analysed in aggregate. Response data were stored securely by the main researcher, with access to the raw dataset restricted to the principal investigator. The consent page informed participants that responses would remain confidential and anonymous but did not specify a data retention period or storage location.
**Development and testing**	State how the survey was developed, including whether the usability and technical functionality of the electronic questionnaire had been tested before fielding.	Sections 2.1 and 2.3. The questionnaire was developed from a review of the relevant literature, and the questionnaire was reviewed for content relevance and clarity by three experts in pharmacology, pharmacotherapy and community pharmacy practice, translated into Arabic and back-translated into English. Pilot testing with eight participants assessed clarity, wording and completion time wording was refined on the basis of this feedback before dissemination. The technical functionality of the electronic form, item display, branching

Checklist item	Explanation	Reported in the manuscript / response
		logic, mandatory-field enforcement, and response capture, was tested prior to fielding.
**Open survey versus closed survey**	An “open survey” is open to any visitor; a closed survey is open only to a known sample (password-protected).	Open survey. The questionnaire link was distributed publicly through social media and institutional channels and was accessible to any eligible visitor without registration or password.
**Contact mode**	Indicate whether initial contact with potential participants was made on the Internet.	Section 2.2. All initial contact was made online; the survey link was disseminated via social media and institutional or professional communication channels.
**Advertising the survey**	How and where was the survey announced or advertised? The wording of the announcement should ideally be published as an appendix.	Section 2.2. The survey was distributed via WhatsApp, Facebook, Twitter/X and Snapchat, together with institutional and professional communication channels. The invitation described the study as a final-year medical student graduation project, stated the estimated completion time (3–5 minutes) and the topic (self-medication with analgesics), and noted that a downloadable leaflet on pain medications would be available at the end of the survey. The full wording is reproduced in Supplementary Material S3, S3.1.
**Web / e-mail**	State the type of e-survey. If an e-mail survey, were responses entered manually or captured automatically?	A web-based questionnaire hosted on Microsoft Forms. Responses were captured automatically by the platform.
**Context**	Describe the website or mailing list on which the survey was posted, and discuss to what degree its content could pre-select the sample.	Section 2.2. No dedicated website was used. The link was shared through general-purpose social media platforms and institutional channels. Because dissemination relied on the investigators’ networks and onward sharing, the resulting sample over-represents younger and university-educated respondents, as reflected in Table 1 and acknowledged in Section 4.
**Mandatory / voluntary**	Was the survey mandatory or voluntary?	Sections 2.2 and 2.7. Participation was entirely voluntary and anonymous.
**Incentives**	Were any incentives offered?	No monetary or material incentives were offered. On completion, all participants were presented with a digital educational brochure on analgesic safety (Supplementary Material S5).
**Time / date**	In what timeframe were the data collected?	Section 2.1. April to October 2024.
**Randomization of items or questionnaires**	To prevent biases, items can be randomized or alternated.	Items were not randomized. The questionnaire followed a fixed order (sociodemographic characteristics, then self-medication practices, then safety knowledge) because later sections depended on earlier responses.

Checklist item	Explanation	Reported in the manuscript / response
**Adaptive questioning**	Use of adaptive questioning — items conditionally displayed based on responses to other items.	Section 2.3. Adaptive questioning was used: participants answering "No" to the self-medication item were not shown the subsequent items on analgesic types, indications, motivations, or safety knowledge. Knowledge items were therefore completed by 332 of 400 participants.
**Number of items**	What was the number of questionnaire items per page?	The questionnaire comprised 14 items: 3 sociodemographic items, 6 items on self-medication practices, and 5 safety knowledge statements. Items per page ranged from 4 to 7.
**Number of screens (pages)**	Over how many pages was the questionnaire distributed?	Four pages; an informed consent page, followed by one page per questionnaire section (sociodemographic characteristics, self-medication practices, and analgesic safety knowledge).
**Completeness check**	Were consistency or completeness checks performed before submission? All items should provide a non-response option.	Section 2.2. All items were configured as mandatory; the form could not be submitted with unanswered items, and consequently no submitted questionnaire contained missing data. Knowledge items included a "Not sure" option, providing a non-response alternative on those items.
**Review step**	Were respondents able to review and change their answers?	Yes
**Unique site visitor**	If view or participation rates are provided, define how a unique visitor was determined.	Not determined. The survey was distributed as an open link across multiple platforms, and unique visitors were not tracked.
**View rate**	Unique visitors to the first survey page divided by unique site visitors.	Not available. Because the survey was distributed as an open link across several social media platforms, the number of individuals who viewed the invitation could not be determined.
**Participation rate**	Unique visitors who agreed to participate divided by unique first survey page visitors.	401 questionnaires were started following 409 accesses of the survey link (98.0%); because the link was open, accesses may not represent unique or eligible individuals.
**Completion rate**	Users who finished the survey divided by users who agreed to participate.	Section 2.2. 400 of 401 who began the questionnaire submitted complete responses (99.8%).
**Cookies used**	Were cookies used to assign a unique user identifier and prevent duplicate entries?	No cookies were used to assign unique identifiers. The survey was open-access and did not require sign-in, so the platform's duplicate-prevention option was not enabled.
**IP check**	Was the IP address used to identify potential duplicate entries?	IP addresses were not recorded or used to identify duplicate entries. Potential duplicates were screened manually during data cleaning (Section 2.6); none were identified.

Checklist item	Explanation	Reported in the manuscript / response
**Log file analysis**	Were other techniques used to identify multiple entries?	Section 2.6. Submitted responses were screened for completeness and potential duplicate entries before analysis; no duplicates were identified.
**Registration**	In closed surveys, describe how duplicate entries from the same user were prevented.	Not applicable; the survey was open-access and did not require registration or login.
**Handling of incomplete questionnaires**	Were only completed questionnaires analysed? Were questionnaires terminated early also analysed?	Section 2.2. Only complete submissions were analysed (n = 400). Because all items were mandatory, questionnaires abandoned before submission were not recorded by the platform and could not be characterized.
**Questionnaires submitted with an atypical timestamp**	Were questionnaires excluded for being submitted too quickly? Specify any cut-off.	No timestamp-based exclusion criterion was applied.
**Statistical correction**	Were weighting or propensity scores used to adjust for a non-representative sample?	Section 2.6. No weighting or propensity adjustment was applied. Estimates are unweighted and describe the sample only; this is stated in Sections 2.2 and 4.

Reference: Eysenbach, G. Improving the quality of Web surveys: the Checklist for Reporting Results of Internet E-Surveys (CHERRIES). J. Med. Internet Res. 2004, 6, e34; erratum J. Med. Internet Res. 2012, 14, e8. Response-rate definitions follow the 2012 erratum.

S3: S3.1 Survey Invitation Message as Distributed

The following invitation was circulated through WhatsApp, Facebook, Twitter/X, Snapchat, and institutional communication channels. Both language versions are reproduced as distributed.

English version

This is a graduation project for final year medical students. We appreciate your participation in this 3–5 min survey on the self-medication of analgesics/pain medication. By the end of the survey, there is a downloadable leaflet about pain medications for your reference.

Arabic version

استبيان لطالبات طب سنة تخرج ... عن الوصف الذاتي للمسكنات الدوائية. وقت الاستبيان ٣ ل ٥ دقائق، جزاكم الله خيراً مقدماً

للعلم يوجد نشرة توعوية عن المسكنات يمكن تنزيلها بآخر الاستبيان للإفادة.

نرجو التكرم بتعبئة الاستبيان

S3: S3.2 SAMPLE SURVEY

We are a group of 5th year medical students and as a part of our curriculum in Dubai Medical College for Girls (DMCG), we are conducting research on Self-Prescription of Analgesics in the UAE: A Cross-Sectional Study

We aim to assess the knowledge, attitude, and practices of self-prescribed pain medication and to evaluate the factors leading to usage among the population of UAE.

الوصفة الذاتية للمسكنات في الإمارات العربية المتحدة :دراسة مقطعية

نجري أبحاثًا حول ،(DMCG) نحن مجموعة من طلاب الطب في السنة الخامسة وكجزء من مناهجنا في كلية دبي الطبية للبنات
المعرفة والمواقف وممارسات استخدام المسكنات بين سكان الإمارات العربية المتحدة.
هدفنا هو تقييم المعرفة والمواقف والممارسات المتعلقة باستخدام المسكنات وتقييم العوامل التي تؤدي إلى استخدامها بين سكان دولة
.الإمارات العربية المتحدة

<u>CONSENT</u>

This questionnaire will take approximately 3-5 minutes to complete, and all information will remain confidential and anonymous. Your participation is voluntary, and no personal information will be disclosed. You may withdraw from this study anytime you wish to do so. All responses are required to be filled.

You can contact the researchers on the below given email for any concerns.

By completing the questionnaire, you give permission to be a part of this study. We highly appreciate your participation and thank you for your valuable time.

موافقة
سيستغرق استكمال هذا الاستبيان ما يقرب من 3-5 دقائق وستظل جميع المعلومات سرية ومجهولة المصدر. مشاركتك تطوعية ولن يتم الكشف عن أي معلومات شخصية. يمكنك الانسحاب من هذه الدراسة في أي وقت ترغب في القيام بذلك. يجب ملء جميع الردود.
يمكنك الاتصال بالباحثين على البريد الإلكتروني أدناه لأي مخاوف.
من خلال إكمال الاستبيان، فإنك تمنح الإذن بأن تكون جزءًا من هذه الدراسة. نحن نقدر بشدة مشاركتك ونشكرك على وقتك الثمين.
مجموعة الباحثين:
عائشة أمل
راية زاهد

سارة فاضل
أنعم منصور
مايا أحمد

Email Id: **aym20190113@dmcg.edu**
Supervisor: Dr. Eslam ElNebrisi

لقد قرأت الموافقة أعلاه وأوافق
نعم / لا

English:

1. Socio-Demographic Data:

- Your gender?
  - Female
  - Male
- Your age?
  - 18-24
  - 25 - 34
  - 35 - 44
  - 45 - 54
  - Above 55
- The highest level of education you have completed?
  - Primary School
  - Secondary School
  - University and above

2. Prevalence of Self Medication among adults in the UAE:

- Have you ever engaged in self-prescription (taking medication without consulting a healthcare professional) of pain medication?
  - Yes
  - No
  - Not sure
- If yes, what type of pain medication(s) have you self-prescribed, among the ones mentioned below? (Select all that apply)
  - Paracetamol (Acetaminophen)
  - Advil (Ibuprofen)
  - Aspirin
  - Voltaren (Diclofenac)
  - Indocin (indomethacin)
  - Others: ------------
- What motivates you to engage in self-prescription? (Select all that apply)
  - Convenience
  - Previous positive experiences
  - Lack of time to visit a healthcare professional.
  - Financial reasons
  - I don’t engage in self-prescription.
- How often do you rely on self-prescription instead of consulting a healthcare professional?
  - Never
  - Occasionally

- Frequently
- Always

➢ What factors influence your choice of pain medication? (Select all that apply)
- Effectiveness in relieving pain
- Personal experience with the medication
- Doctor's recommendation
- Concerns about side effects
- Affordability
- Ease of accessibility
- None of the above

➢ What are the primary reasons or conditions for which you typically use analgesics (pain relievers)? Please select the one main reason from the options below:
- Headache
- Fever
- Menstrual pain
- Muscular pain
- Joint pain or arthritis
- Dental pain
- Back pain
- Post surgery pain

➢ What action do you take when self-medication fails?
- Seeking medical consultation
- Waiting for symptoms to resolve on its own
- Double dosing (increasing the dose of the same medicine)
- Repeating the same drug
- Using a stronger (different) drug
- Using traditional medicine

3. Knowledge of Toxicity

Knowledge about side effects of NSAIDs and paracetamol.	True	False	Not sure
NSAIDs can cause irritation and damage to the lining of the stomach and intestines			
NSAIDs are generally safe for individuals with kidney problems			
Paracetamol (acetaminophen) has no risk of toxicity, even at high doses.			
Excessive or prolonged use of paracetamol can lead to severe liver damage			
In rare cases, paracetamol can cause allergic reactions, causing skin rash, swelling, etc.			

Arabic:

1. **البيانات الاجتماعية والديموغرافية**

➢ جنسك؟
- أنثى
- ذكر

- عمرك؟
  - ١٨-٢٤
  - ٢٥-٣٤
  - ٣٥-٤٤
  - ٤٥-٥٤
  - أكثر من ٥٥

- أعلى مستوى تعليم أكملته؟
  - المدرسة الابتدائية
  - المدرسة الثانوية
  - الجامعة وأكثر

2. **انتشار التداوي الذاتي بين البالغين في الإمارات:**

- هل سبق لك التداوي ذاتيا (تناول الأدوية بدون استشارة الطبيب) بمسكن الألم؟
  - نعم
  - لا
  - غير متأكد

- إذا كانت الإجابة نعم، أي نوع من أنواع مسكنات الألم قمت باستعماله؟ (اختر كل ما ينطبق)
  - باراسيتامول (أسيتامينوفين)
  - أدفيل (ايبوبروفين)بروفين
  - . أسبرين
  - فولتارين (ديكلوفيناك)
  - إندوسين (إندوميثاسين)
  - أخرى: ----------------

- ما الذي يدفعك للتداوي الذاتي؟ (اختر كل ما ينطبق)
  - الشعور بالراحة
  - تجارب إيجابية سابقة (مثلا: التخلص من الألم)
  - عدم وجود وقت لزيارة الطبيب
  - أسباب مالية
  - لا أتداوى بشكل ذاتي

- كم مرة تعتمد على التداوي الذاتي بدلاً من استشارة الطبيب؟
  - أبدًا
  - بشكل عرضي / قليل جدا
  - بشكل متكرر
  - . دائمًا

- ما هي العوامل التي تؤثر في اختيارك لمسكن الألم؟ (اختر كل ما ينطبق)
  - فعالية الدواء في تخفيف الألم
  - التجربة الشخصية مع الدواء

- توصية سابقة من طبيب
- مخاوف من الآثار الجانبية للمسكنات الأخرى
- التكلفة المادية
- سهولة الحصول على الدواء
- لا شيء من ذلك

- ما هي الأسباب الرئيسية أو الظروف التي تستخدم فيها مسكنات الألم عادة؟ يرجى اختيار السبب الرئيسي واحد من الخيارات التالية
  - الصداع
  - الحمى (ارتفاع درجة الحرارة لأي سبب)
  - آلام الحيض
  - الآلام العضلية
  - آلام المفاصل أو التهاب المفاصل
  - آلام الأسنان.
  - آلام الظهر
  - آلام بعد الجراحة

- ما الإجراء الذي تتخذه عندما يفشل التداوي الذاتي؟
  - استشارة طبيب مختص
  - الانتظار حتى تتحسن الأعراض بشكل طبيعي
  - زيادة الجرعة (زيادة جرعة نفس الدواء)
  - تكرار نفس الدواء
  - استخدام مسكن أقوى أو مختلف
  - استخدام الطب البديل

3. **معرفة السمية**

**المعرفة حول الآثار الجانبية لمضادات الالتهاب اللاستيرويدية غير الستيرويدية (NSAIDs) والباراسيتامول**	**صحيح**	**خاطئ**	**غير متأكد**
**مضادات الالتهاب غير الستيرويدية (NSAIDs) يمكن أن تسبب تهيجًا وضررًا لبطانة المعدة والأمعاء.**			
**مضادات الالتهاب غير الستيرويدية (NSAIDs) بشكل عام غير آمنة للأفراد الذين يعانون من مشاكل في الكلى.**			
**الباراسيتامول (الأسيتامينوفين) ليس لديه خطر سمية، حتى عند تناول جرعات عالية.**			
**استخدام الباراسيتامول بشكل زائد أو لفترات طويلة يمكن أن يؤدي إلى تلف كبير في الكبد.**			
**في حالات نادرة، يمكن أن يسبب الباراسيتامول تفاعلات تحسسية تسبب طفح جلدي وتورم وما إلى ذلك.**			

Supplementary Material S4

Educational brochure on analgesic safety, as distributed to participants

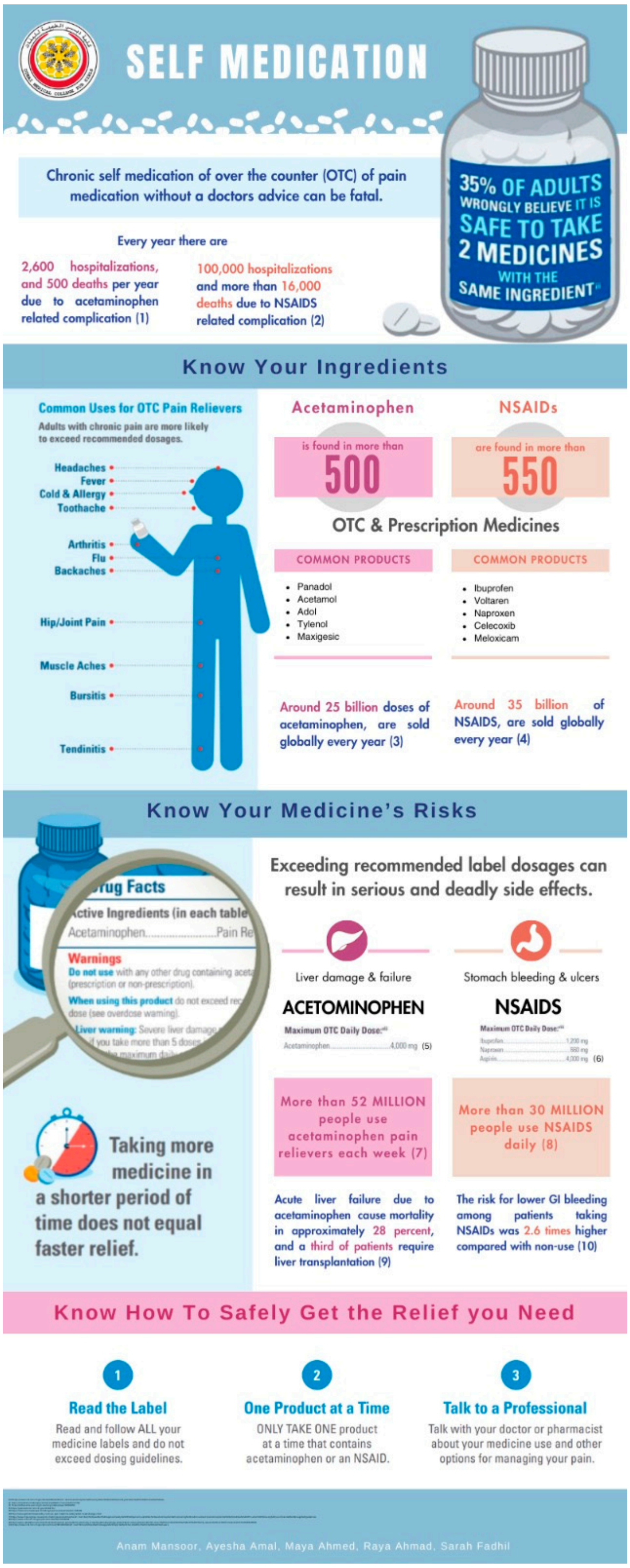

Supplementary Material S5

Post Hoc Psychometric Evaluation of the Study-Specific Five-Item Analgesic Safety Knowledge Index

Manuscript: *Analgesic Self-Medication and Safety Knowledge Among Adults in the United Arab Emirates: A Cross-Sectional Online Survey*

S5.1. Purpose and Scope

The five-item analgesic safety knowledge index used in this study was developed for the present survey rather than adapted from a previously validated instrument. Because the index was not validated before administration, its measurement properties were evaluated post hoc using the study data. These analyses are reported here in full for transparency; the corresponding summary statements appear in Section 2.3 of the main manuscript.

All analyses are based on the 332 participants who reported analgesic self-medication and who therefore completed the knowledge items. Items were scored dichotomously (1 = correct; 0 = incorrect or "Not sure"), and the five items were summed to yield a composite score ranging from 0 to 5. In the Arabic version, item 2 was phrased negatively, and Arabic-version responses to this item were scored accordingly (Section 2.3 of the main manuscript). Analyses were conducted in IBM SPSS Statistics (version 28.0) and Python (version 3).

These results should be interpreted as post hoc evidence that the index behaved coherently in this sample, not as formal validation of a measurement instrument. Establishing validity would require independent administration in a separate sample, together with assessment of test–retest reliability and criterion or construct validity against an external standard — none of which the present cross-sectional design permits.

S5.2. Item Analysis and Internal Consistency

Item analysis of the analgesic safety knowledge index (n = 332)

Item	p	M	SD	r_{it}	D	α if deleted
1. NSAIDs cause gastrointestinal damage	.702	0.70	0.46	.514	.752	.648
2. NSAIDs safe in kidney disease (F)	.560	0.56	0.50	.491	.889	.656
3. Paracetamol non-toxic at high doses (F)	.675	0.67	0.47	.435	.749	.679
4. Paracetamol causes liver damage	.711	0.71	0.45	.506	.757	.652
5. Paracetamol causes allergic reactions	.473	0.47	0.50	.412	.899	.690

Note. p = item difficulty (proportion answering correctly). M and SD are based on binary scoring. r_{it} = corrected item–total correlation. D = discrimination index (upper 27% minus lower 27%; cases tied at the group boundaries were apportioned proportionally). α if deleted = Cronbach's alpha with the item removed. Overall α = .713; standardised α = .715; mean inter-item r = .334. (F) denotes items for which the correct response is "False".

Internal consistency was acceptable (Cronbach's α = .713), meeting the conventional threshold of .70 for research instruments [1]. The standardised alpha (.715) closely approximated the raw coefficient, and the

mean inter-item correlation (.334) fell within the range of .15–.50 recommended for scales measuring a broad construct [2], indicating that items were related without being redundant.

Item difficulty ranged from .473 (paracetamol allergy) to .711 (paracetamol liver damage), placing all items in the moderate range. All corrected item–total correlations exceeded .40 (range .412–.514), and discrimination indices ranged from .749 to .899, exceeding the .40 benchmark. Removal of any single item would have reduced alpha (range .648–.690), indicating that each item contributed positively to internal consistency.

S5.3. Inter-Item Correlations

Inter-item correlation matrix (n = 332)

Item	1	2	3	4	5
1. NSAIDs gastrointestinal damage	—	.417	.292	.325	.393
2. NSAIDs kidney disease (F)		—	.343	.345	.280
3. Paracetamol toxicity (F)			—	.422	.194
4. Paracetamol liver damage				—	.325
5. Paracetamol allergy					—

Note. Pearson correlations on binary-scored items. All $p < .001$.

Inter-item correlations ranged from .194 (paracetamol toxicity with allergy) to .422 (paracetamol toxicity with liver damage). No correlation exceeded .80, indicating no redundancy, and none was negative, which is consistent with unidimensionality. The strongest association linked the two paracetamol dose-related items, consistent with their shared content domain. The weakest pairings involved the allergy item, suggesting it may capture a partially distinct dimension — a pattern examined further below.

S5.4. Factor Structure

Principal component analysis of the analgesic safety knowledge index (n = 332)

	**Unrotated solution**	**Varimax rotated**		
**Item**	**Loading**	***h*²**	**Component 1**	**Component 2**
1. NSAIDs gastrointestinal damage	.719	.516	.291	.737
2. NSAIDs kidney disease (F)	.705	.498	.531	.465
3. Paracetamol toxicity (F)	.653	.426	.875	.028
4. Paracetamol liver damage	.713	.509	.708	.290
5. Paracetamol allergy	.626	.391	.057	.848
**Eigenvalue**	2.340		0.860	
**Variance explained**	46.8%		17.2%	

Note. h^2 = communality. Bartlett's test of sphericity: $\chi^2(10) = 281.47$, $p < .001$. Kaiser–Meyer–Olkin measure = .755. The Kaiser criterion retained one component; the two-component varimax solution is shown for interpretive comparison only. Split-half reliability (Spearman–Brown corrected) = .740.

The data were suitable for factor analysis: Bartlett's test of sphericity was significant ($\chi^2(10) = 281.47$, $p < .001$) and the Kaiser–Meyer–Olkin measure was .755, exceeding the .60 threshold. Principal component analysis yielded a single component with an eigenvalue of 2.340, explaining 46.8% of total variance and the only component exceeding the Kaiser criterion. All items loaded on this component between .626 and .719, supporting interpretation as a single knowledge construct. Communalities ranged from .391 (allergy) to .516 (gastrointestinal damage).

A two-component varimax solution was examined for interpretive transparency. Component 1 grouped the three dose- and organ-toxicity items (the kidney item loading moderately on both components), while Component 2 loaded on the gastrointestinal and allergy items. Given the clear separation of eigenvalues (2.340 versus 0.860) and the single-component Kaiser solution, the unidimensional interpretation was retained. Split-half reliability (Spearman–Brown corrected) was .740.

Because Pearson (phi) correlations between binary items are constrained by item difficulty and can understate the underlying association, the principal component analysis was repeated on the matrix of tetrachoric correlations as a sensitivity analysis. Tetrachoric correlations ranged from .314 (paracetamol toxicity with allergy) to .636 (gastrointestinal damage with kidney disease). The first component had an eigenvalue of 3.09 and explained 61.9% of the variance; the second component had an eigenvalue of 0.76, below the Kaiser criterion. Loadings on the first component ranged from .732 (allergy) to .828 (gastrointestinal damage). This analysis supports the same single-component interpretation. In both analyses, principal component analysis was used descriptively, to check whether summing the five items into one score was reasonable, and not as evidence of construct validity.

S5.5. Summary

The five-item index demonstrated acceptable internal consistency, adequate item discrimination, and a predominantly unidimensional structure in this sample. These properties support its use as a brief composite measure of selected analgesic safety concepts in the present analysis. As noted in Section S4.1, the index has not been independently validated, and comparison of absolute scores with those from other instruments is not appropriate.

References

1. Nunnally, J.C.; Bernstein, I.H. *Psychometric Theory*, 3rd ed.; McGraw-Hill: New York, NY, USA, 1994.

2. Clark, L.A.; Watson, D. Constructing validity: Basic issues in objective scale development. *Psychol. Assess.* 1995, 7, 309–319.

Supplementary Material S6

Sensitivity analysis of age coding in the multivariable models of analgesic self-medication (N = 400) and safety knowledge (n = 332)

Adjusted estimates for sex and age under four codings of age.

Age coding	Model 1: female aOR (95% CI)	Model 1: age	Model 2: female B (95% CI)	Model 2: age	Model 3: female aOR (95% CI)	Model 3: age
Midpoint, per year (primary)	2.08 (1.12–3.86)	aOR 1.02 (0.99–1.04)	0.55 (0.15–0.95)	B −0.027 (−0.041 to −0.012)	2.21 (1.29–3.77)	aOR 0.97 (0.95–0.99)
Ordinal (1–5)	2.07 (1.12–3.85)	aOR 1.19 (0.94–1.51)	0.53 (0.14–0.93)	B −0.28 (−0.42 to −0.13)	2.17 (1.27–3.71)	aOR 0.72 (0.60–0.88)
Categorical (ref 18–24)	1.96 (1.05–3.66)	See Table S6.2	0.53 (0.13–0.94)	See Table S6.2	2.22 (1.29–3.84)	See Table S6.2
Binary (≥35 vs 18–34)	1.94 (1.07–3.50)	aOR 1.48 (0.81–2.71)	0.65 (0.27–1.04)	B −0.64 (−1.01 to −0.28)	2.42 (1.45–4.06)	aOR 0.43 (0.27–0.71)

aOR, adjusted odds ratio; B, unstandardized regression coefficient; CI, confidence interval. All models were adjusted for sex, age and education (ordinal). Model 1: binary logistic regression for lifetime self-medication (N = 400). Model 2: linear regression of the 0–5 knowledge score (n = 332). Model 3: binary logistic regression for adequate knowledge, ≥4/5 correct (n = 332). The midpoint row reproduces Table 8. Categorical coding did not improve model fit over the midpoint term in any model (Model 1: likelihood-ratio $\chi^2(3) = 4.05$, $p = 0.257$; Model 2: $F(3, 325) = 0.64$, $p = 0.591$; Model 3: likelihood-ratio $\chi^2(3) = 2.82$, $p = 0.420$).

Full estimates from the models with age entered as categorical indicators (reference: 18–24 years).

Predictor	n (Model 1 / Models 2–3)	Model 1: aOR (95% CI)	p	Model 2: B (95% CI)	p	Model 3: aOR (95% CI)	p
18–24 (reference)	144 / 119	1.00	—	0.00	—	1.00	—
25–34	58 / 45	0.79 (0.35–1.76)	0.560	−0.35 (−0.91 to 0.20)	0.214	0.73 (0.35–1.53)	0.403
35–44	103 / 84	1.00 (0.48–2.10)	0.998	−0.68 (−1.15 to −0.22)	0.004	0.38 (0.20–0.71)	0.003
45–54	60 / 55	2.72 (0.91–8.11)	0.073	−0.77 (−1.31 to −0.23)	0.005	0.45 (0.22–0.92)	0.030
≥55	35 / 29	1.43 (0.47–4.38)	0.532	−1.13 (−1.84 to −0.42)	0.002	0.27 (0.10–0.76)	0.013
Female		1.96 (1.05–3.66)	0.034	0.53 (0.13 to 0.94)	0.010	2.22 (1.29–3.84)	0.004
Education level		1.69 (0.85–3.34)	0.131	0.32 (−0.18 to 0.81)	0.207	1.72 (0.89–3.33)	0.105

aOR, adjusted odds ratio; B, unstandardized regression coefficient; CI, confidence interval. Each model includes the four age indicators, sex (female vs. male) and education (ordinal). Model 1 includes all 400 participants; Models 2 and 3 include the 332 self-medicating participants. Mean knowledge scores by age group (Models 2–3 sample) were 3.65 (18–24 years), 3.20 (25–34), 2.90 (35–44), 2.73 (45–54) and 2.21 (≥55).
